# Understanding the Variability of Peanut‐Oral Immunotherapy Responses by Multi‐Omics Profiling of Immune Cells

**DOI:** 10.1111/all.16627

**Published:** 2025-07-22

**Authors:** Aleix Arnau‐Soler, Sarah E. Ashley, Ahla Ghauri, Alexander C. S. N. Jeanrenaud, Ingo Marenholz, Katharina Blumchen, Penelope Cibin, Alisa Iakupova, Norbert Hubner, Kirsten Beyer, Young‐Ae Lee

**Affiliations:** ^1^ Max‐Delbrück‐Center for Molecular Medicine Berlin Germany; ^2^ Clinic for Pediatric Allergy Experimental and Clinical Research Center of Max‐Delbrück‐Center for Molecular Medicine and Charité‐Universitätsmedizin Berlin Berlin Germany; ^3^ German Center for Child and Adolescent Health (DZKJ) partner site Berlin Berlin Germany; ^4^ Division of Pneumology, Allergology, Infectious Diseases and Gastroenterology, Department of Pediatrics Goethe University Frankfurt Frankfurt am Main Germany; ^5^ Department of Pediatric Respiratory Medicine, Immunology, and Intensive Care Medicine Charité‐Universitätsmedizin Berlin Berlin Germany

**Keywords:** biomarkers, DNA methylome, oral immunotherapy, peanut allergy, transcriptome

## Abstract

**Background:**

Oral immunotherapy (OIT) induces desensitization in peanut allergy, yet 15%–30% of patients do not respond, and a significant risk of anaphylaxis due to treatment remains. In a placebo‐controlled peanut OIT trial, this study identifies molecular drivers of OIT responsiveness through multi‐omics profiling in immune cells.

**Methods:**

Immunoglobulins, cytokines, transcriptome, and DNA methylome profiles were analyzed in peanut‐stimulated and unstimulated peripheral blood mononuclear cells isolated from peanut‐allergic children before and after treatment. Multi‐omics profiling focused on OIT responsiveness within the active treatment arm. Additional subgroup analyses were performed to further elucidate molecular mechanisms and potential biomarkers.

**Results:**

Complete responders, tolerating 4500 mg of peanut protein, exhibited lower pre‐treatment peanut‐specific IgE and Th2 cytokine production (IL‐4, IL‐5) compared to incomplete responders who tolerated ≤ 1000 mg of peanut protein after treatment. Our primary analysis identified 184 differentially expressed genes and 1001 differentially methylated genes, enriched for innate (ILC3) and adaptive (CD8αα subset of CD8^+^ T cells) immune cells, alongside γδ T cells and exosomes, highlighting gastrointestinal regulatory processes as central to OIT success. We found a marked downregulation of immunoglobulin genes in patients receiving peanut compared to placebo, suggesting OIT‐induced modulation of B‐cell activity. Functional networks revealed a marked imbalance contrasting regulatory T‐cell responses and B‐cell suppression in the complete responders with innate immune signaling and metabolic stress in the incomplete responders.

**Conclusion:**

This multi‐omics approach underscores the importance of gastrointestinal immune mechanisms underlying the variation in peanut oral immunotherapy responses and offers potential biomarkers for improving treatment strategies.

**Trial Registration:**

German Clinical Trials Register DRKS00004553

## Graphical abstract

This study analyzes molecular drivers of OIT responsiveness. Multi‐omics profiling of peanut‐stimulated PBMCs in a controlled peanut‐OIT trial identifies immune, transcriptomic, and epigenetic changes distinguishing complete responders from incomplete responders. Key findings highlight gastrointestinal immune mechanisms, including extracellular exosome processes and imbalanced immune responses: regulatory T‐cell dominance and B‐cell suppression in responders versus myeloid inflammation, metabolic stress, and elevated Th2 cytokines and IgE in non‐responders. DEGs, differentially expressed genes; DNAm, DNA methylation; DMRs, differentially methylated regions; IgE, immunoglobulin E; ILC3, group 3 innate lymphoic cell; OFC, oral food challenge; OIT, oral immunotherapy; PBMCs, peripheral blood mononuclear cells; RNAseq, RNA sequencing; RUNX, runt‐related transcription factors; STAT6, signal transducer and activator of transcription 6; Th2, T helper type 2.

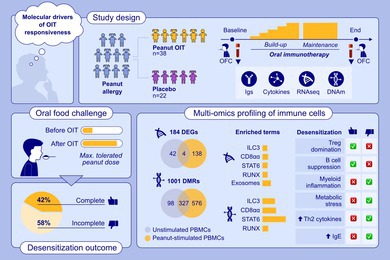

## 1 Introduction

Peanut allergy is a growing public health concern in industrialized countries affecting up to 3% of children [1, 2] and generally persisting into adulthood. Peanut is a major elicitor of severe anaphylactic reactions [3, 4] and food‐allergy‐related deaths [5]. Oral immunotherapy (OIT) aims to desensitize allergic patients through daily ingestion of increasing peanut doses. While clinical trials show efficacy in increasing tolerance thresholds in most patients [6, 7], 15%–30% do not achieve desensitization [8, 9], and side effects remain a concern: in a recent review of 783 patients receiving peanut OIT in a private practice setting, a sizeable proportion of 10% reported systemic allergic reactions during up‐dosing, and 19% during maintenance, often requiring epinephrine (47%) and medical attention (57%) [10]. Given these challenges, a personalized approach is essential to minimize risk and improve patient care.

**FIGURE 1:**
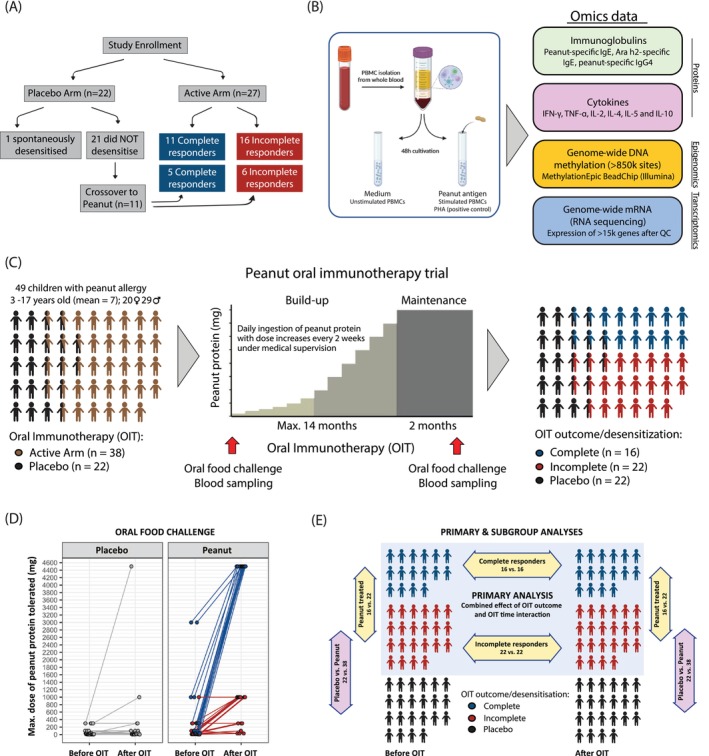
Study design. (A) Study recruitment and classification of participants. Of 49 children with peanut allergy enrolled in the trial, 27 were randomly allocated to the active arm and received oral immunotherapy (verum), while 22 received placebo. At the end of the trial, 11 placebo participants crossed over and received verum treatment. Based on the maximum tolerated dose of peanut protein after OIT, 16 participants were classified as complete responders and 22 as incomplete. (B) Experimental workflow. Blood samples were collected before and after OIT. Serum was used to measure immunoglobulin levels. PBMCs were isolated and either stimulated with peanut extract, PHA, or maintained in medium for 48 h. Cytokine levels were measured in the culture supernatant. Genome‐wide DNA methylation profiling and mRNA sequencing were performed on cultured PBMC samples. (C) Final classification of participants based on OIT response, including placebo and crossover groups. A total of 49 children with peanut allergy (mean age = 7 years) were enrolled. Each child underwent an OFC before and after treatment. At the end of the trial, 16 were classified as complete responders (blue, tolerating 4500 mg of peanut protein), and 22 as incomplete responders (red, tolerating ≤ 1000 mg). (D) Individual changes in maximum tolerated dose of peanut protein from the initial to the final OFC, shown for verum participants receiving peanut: Complete responders (blue), incomplete responders (red), and placebo participants (gray). (E) Overview of all comparisons performed in the study. Diagram summarizing the primary analysis (combined effect of OIT outcome and its interaction with time) and all subgroup analyses across time points, treatment arms, and stimulation conditions. IFN‐γ, interferon gamma; IgE, immunoglobulin E; IgG4, immunoglobulin G4; IL‐10, interleukin 10; IL‐2, interleukin 2; IL‐4, interleukin 4; IL‐5, interleukin 5; OFC, oral food challenge; OIT, oral immunotherapy; PBMCs, peripheral blood mononuclear cells; PHA, phytohemagglutinin; TNF‐α, tumor necrosis factor alpha.

OIT has been shown to induce broad immune modulation across multiple cell types. Studies have reported shifts in allergen‐specific CD4 T cells toward less inflammatory phenotypes [11], reductions in Th2‐associated responses [12], and changes in gut and peripheral immune compartments [13, 14]. There is a substantial gap in understanding the molecular mechanisms underlying the variation in the peanut OIT response. Longitudinal analysis of molecular changes occurring during OIT and their correlation with treatment responses could provide mechanistic insights and potentially reveal predictors of OIT effectiveness. Our study implements a multi‐omics approach, using transcriptomics and epigenomics data with clinical and cellular measures to identify biomarkers associated with OIT outcomes in children with peanut allergy [9] (Figure 1A–C). Using standardized titrated oral food challenges before and after OIT, we quantified the treatment response (Figure 1D) and analyzed peripheral blood mononuclear cells (PBMCs) with and without allergen‐specific stimulation. By combining genome‐wide gene expression and DNA methylation profiles, we identified differentially expressed and methylated genes associated with the strength of the OIT response. Enrichment analyses highlighted specific immune cell types and mechanisms mainly in the gut. Our results elucidate the molecular basis of the variability in peanut OIT responses and provide candidate biomarkers for predicting OIT outcomes, advancing toward personalized OIT strategies.

## 2 Methods

Please see the Online Repository for full details on clinical phenotyping, transcriptomic, and epigenomic data generation, experimental, and analytical methods.

### 2.1 Study Design and Participants

The study design is summarized in Figure 1. We investigated 49 children (29 boys, 20 girls; mean age = 7.0 years) with IgE‐mediated, challenge‐proven peanut allergy who have participated in a double‐blind, randomized placebo‐controlled peanut OIT trial as previously described [9]. Briefly, 27 study participants received peanut and 22 received placebo. 11 placebo children subsequently received peanut, resulting in a final group of 38 peanut‐allergic children in the active arm (Figure 1A). Participants in the active arm underwent dose increases up to the target dose of 125–250 mg of peanut protein for up to 14 months and maintained that dose for another 2 months. The study was approved by the Ethics Commission of Charite‐Universitätsmedizin Berlin, and written informed consent was provided by all caregivers.

### 2.2 Procedures and Data Collection

Participants underwent titrated oral food challenges (OFC) with roasted peanuts before and after OIT to determine the individual peanut dose eliciting an allergic response [15] using a modified PRACTALL protocol [16]. Blood samples were collected before the initial and final OFC. A skin prick test (SPT) was performed as a prick‐to‐prick test with the natural, roasted whole peanut. Serum levels of peanut‐ and Ara h 2‐specific IgE and peanut‐specific IgG4 were measured (CAP100 System FEIA, Thermo Fisher). PBMCs were isolated and cultivated in medium, with peanut extract (50 μg/mL) or with phytohemagglutinin (PHA, 20 μg/mL) as a positive control for 48 h, then subjected to co‐extraction of DNA and RNA. RNA sequencing on a HiSeq 4000 and cytokine analysis (IFN‐γ, TNF‐α, IL‐2, IL‐4, IL‐5, and IL‐10) in the PBMC culture supernatants [15] were performed for all conditions, while DNA methylation profiling using the Infinium Methylation EPIC BeadChip was conducted only in unstimulated and peanut‐stimulated PBMCs.

### 2.3 Oral Immunotherapy Outcome Definition

After treatment, participants were classified at the final OFC as either complete responders if tolerating the highest dose of 4500 mg peanut protein or incomplete responders if tolerating ≤ 1000 mg (Figure 1D). Additionally, we used the difference in tolerated peanut protein between the initial and the final OFC as a quantitative trait to analyze the magnitude of tolerance improvement (Figure S1).

### 2.4 Statistical Analyses

To investigate the association of serum immunoglobulin and cytokine levels in PBMC culture supernatants with the OIT response, we performed pairwise comparisons using Wilcoxon's signed‐rank tests and Kruskal‐Wallis analysis. Results were corrected for multiple testing.

Transcriptomic and epigenomic analyses were performed in PBMCs composed of multiple immune cell populations. To adjust for cell type heterogeneity, we used Scaden [17], a deep‐learning‐based deconvolution tool trained on simulated bulk data from single‐cell RNA sequencing (Appendix S1).

To identify differentially expressed genes (DEGs) associated with a stronger OIT response, we applied a likelihood ratio test to compare two models: a full model including sample collection time (pre‐ or post‐OIT), OIT outcome (binary or quantitative) and their interaction; and a reduced model excluding OIT outcome and the interaction term. This approach captured genes with dynamic changes due to time and treatment outcome interaction, as well as those consistently differing across treatment groups. Both models accounted for age, sex, RNA integrity number, PBMC composition (CD4+, CD8+, other T cells, B‐cells, natural killer cells, monocytes, dendritic cells, and others), and one factor of unwanted variation.

To further elucidate molecular mechanisms and potential biomarkers, we performed additional subgroup analyses (Figure 1E) in (1) pre‐OIT samples, comparing complete vs. incomplete responders and peanut versus placebo participants, and (2) post‐OIT samples with the same comparisons. Finally, we analyzed the changes observed before versus after OIT within each group of (3) complete and (4) incomplete responders. For subgroup analyses, we applied a Wald test in the corresponding models, as described in the supplemental materials. *P‐*values were adjusted for multiple testing using a False Discovery Rate (FDR) of 0.1. To understand gene expression changes beyond the individual gene level, we performed a Weighted Gene Co‐expression Network Analysis (WGCNA, Appendix S1) for each subanalysis 1–4. WGCNA was conducted in both unstimulated and peanut‐stimulated transcriptome data. To identify differentially methylated probes (DMPs), DNA methylation data was analyzed using Meffil in the same comparisons detailed above for gene expression [18]. Covariate effects were adjusted, and the genome‐wide significance threshold was set to 9 × 10^−8^ [19]. We validated the significance of our findings by conducting 1000 epigenome‐wide association analyses on randomly permuted phenotypes (Figure S2). Differentially methylated regions (DMRs) were identified using Comb‐p [20] with a seed *p‐*value of 0.05, a maximum distance of 750 bp, and a minimum of 3 CpG probes. Statistical significance (*p* < 0.05) was determined after Sidak correction for multiple testing.

To detect expression quantitative trait methylation (eQTM), we tested for correlation between the degree of DNA methylation at significant DMPs and the expression level of significant nearby DEGs using Kendall's correlation, with *p <* 0.05 after FDR adjustment.

To identify biological processes associated with transcriptional and epigenetic changes, we performed functional enrichment analysis with Enrichr [21] for cell types, diseases/drugs, ontologies, pathways, and transcription factors. The analysis was conducted for all significant genes associated with a stronger OIT response, and separately for genes identified under peanut‐stimulated and unstimulated conditions. Empirical *p*‐values from 1000 enrichment permutations confirmed the robustness of the analyses from both unstimulated and peanut‐stimulated PBMCs. Functional enrichment analyses were also used to annotate co‐regulated genes detected by WGCNA. Criteria for significance included terms with ≥ 10 genes and ≥ 3 differentially expressed/methylated genes, with an FDR‐adjusted significance threshold of *p <* 0.05. Hypergeometric tests were used to detect significant overlaps between: differentially expressed and methylated genes; and immune cells detected via transcriptome and DNA methylome profiling.

## 3 Results

Of 38 peanut‐allergic children in the active arm of the trial, 16 tolerated the final largest dose of 4500 mg peanut protein during the exit challenge (complete responders), whereas the remaining 22 tolerated ≤ 1000 mg (incomplete responders, Figure 1E). Complete responders had a significantly higher initial eliciting and maintenance dose compared to incomplete responders (Table S1). Initial eliciting and maintenance doses were significantly correlated (*R* = 0.503, *p*‐value = 0.0021). Demographical and immunological baseline characteristics of all participants, including placebos, are shown in Table S1.

### 3.1 Lower Pre‐OIT IgE and Cytokine Levels Improve Treatment Success

**FIGURE 2:**
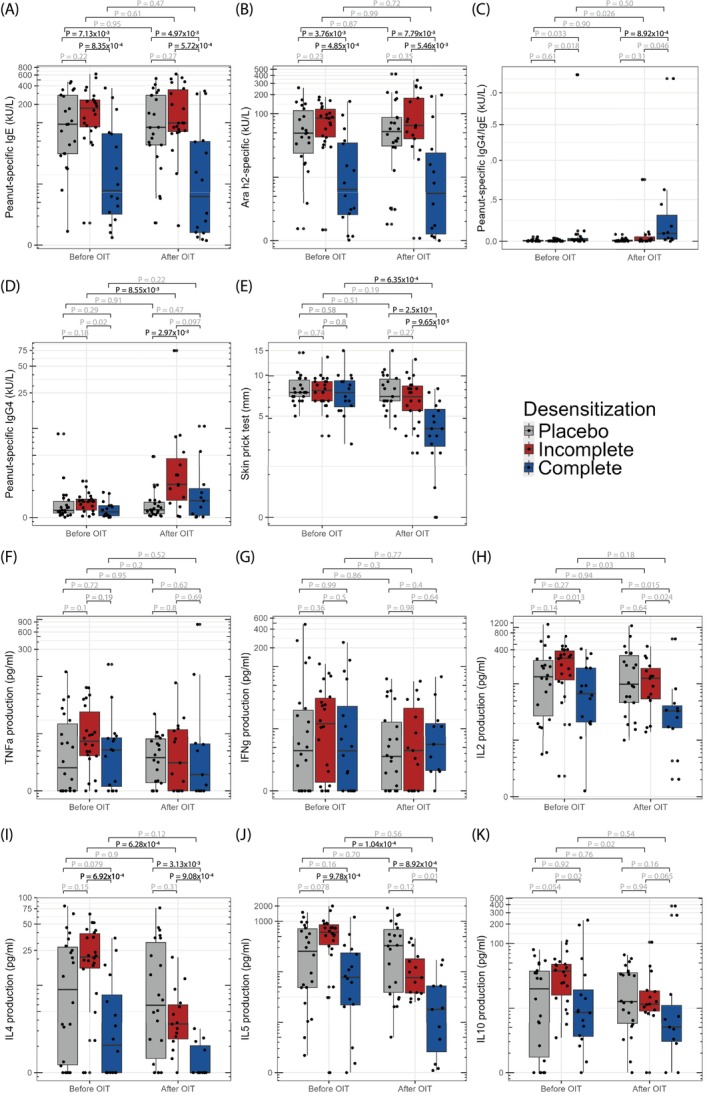
Immunological markers and peanut‐induced cytokines in PBMC culture supernatants before and after OIT. (A–D) Serum immunoglobulin responses: (A) Peanut‐specific IgE, (B) Ara h 2‐specific IgE, (C) peanut‐specific IgG4/IgE ratio, and (D) peanut‐specific IgG4. (E) Skin prick test reactivity to peanut extract, measured as wheal diameter (mm). (F–K) Cytokine responses in PBMC culture supernatants after 48 h of peanut stimulation, adjusted by subtracting cytokine levels from unstimulated (medium‐only) PBMC cultures: (F) TNF‐α, (G) IFN‐γ, (H) IL‐2, (I) IL‐4, (J) IL‐5, and (K) IL‐10. Data are shown for 49 participants: 16 complete responders (blue), 22 incomplete responders (red), and 22 placebo participants (gray). For 11 participants who crossed over to the active arm after initially receiving placebo, post‐OIT data were not available for Ara h 2‐specific IgE, peanut‐specific IgG4, SPT, and cytokine levels; thus, post‐OIT comparisons include 11 complete and 16 incomplete responders. All values are displayed on a logarithmic scale where applicable. Non‐significant *p*‐values after correction for multiple testing (*p* < 0.01 for A–E; *p* < 0.0083 for F–K) are indicated in gray. Wilcoxon signed‐rank tests with continuity correction were used for within‐group comparisons (pre‐ Vs. post‐OIT), and Kruskal–Wallis one‐way analysis of variance on ranks was used for between‐group comparisons. Box plots represent the median (central line), interquartile range (box), and 5th to 95th percentiles (whiskers). IFN‐γ, interferon gamma; IgE, immunoglobulin E; IgG4, immunoglobulin G4; IL, interleukin; OIT, oral immunotherapy; PBMCs, peripheral blood mononuclear cells; SPT, skin prick test; TNF‐α, tumor necrosis factor alpha.

Levels of peanut‐ and Ara h 2‐specific IgE were significantly lower in complete compared to incomplete responders (Figure 2A,B). This difference was already present before OIT, suggesting that higher peanut‐specific IgE levels may reduce OIT responsiveness. Indeed, no significant change in IgE levels was observed after treatment within either group. Contrarily, peanut‐specific IgG4, known as a blocker of IgE effects and thus a potential marker for successful OIT responses [22], was initially low in both groups but increased significantly during OIT only in incomplete responders. The peanut‐specific IgG4 to IgE ratio showed an increase in complete responders that was, however, not significant. The peanut skin prick wheal size decreased significantly after OIT in complete versus incomplete responders (Figure 2C–E).

Cytokine analysis showed no difference in IFN‐γ, TNFα, IL‐2 and IL‐10 between groups (Figure 2F–H). However, higher pre‐OIT levels of Th2 cytokines IL‐4 and IL‐5 were associated with incomplete desensitization. Interestingly, IL‐4 and IL‐5 levels in incomplete responders significantly decreased upon OIT, approaching the baseline levels observed in complete responders (Figure 2I–K). Similar results were obtained using raw cytokine values Figures S3–S6.

### 3.2 Expression of 184 Genes Is Associated With the OIT Response

RNA sequencing of immune cells identified changes in gene expression associated with the variation in OIT responses. We tested the binary outcome “complete versus incomplete responders” and the quantitative outcome “increase in tolerated peanut protein after OIT” (Figure S1). To capture allergen‐specific differences, analyses were performed in unstimulated and peanut‐stimulated PBMCs (Figure 1B). No significant differences in cell type numbers were observed between desensitization groups in unstimulated or peanut‐stimulated PBMCs at the first (8 cell types) or the second (28 cell types) resolution (Figure S7).

**FIGURE 3:**
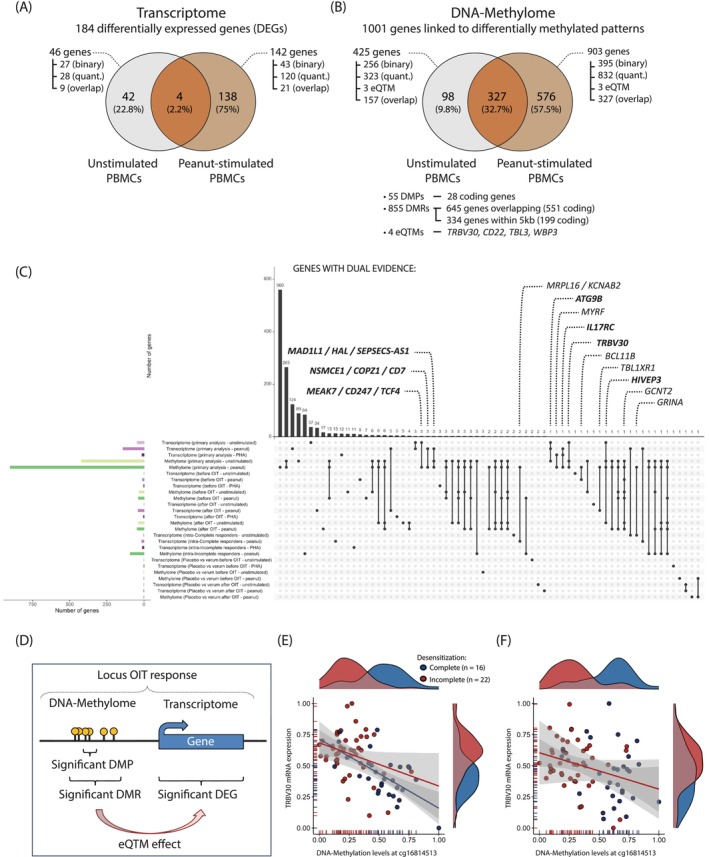
Transcriptomic and epigenomic signatures associated with the peanut OIT response. (A) Number of DEGs identified from PBMC transcriptome data after 48 h of culture under unstimulated (gray) or peanut‐stimulated (brown) conditions. Within each condition, DEGs were detected in association with either the binary outcome (*complete* Vs. *incomplete responders*) or the quantitative trait (*increment in tolerated peanut protein*). The overlap of DEGs for the binary and quantitative phenotype is also shown. (B) Number of genes linked to differentially methylated patterns, including DMPs, DMRs, or eQTM sites, under the same stimulation conditions and phenotype comparisons as in (A). (C) UpSet plot showing the number of genes identified across primary and subgroup analyses of the transcriptome (green bars) and methylome (purple bars) under three stimulation conditions: Unstimulated (light shade), peanut‐stimulated (medium shade), and PHA‐stimulated PBMCs (dark shade). Bars represent genes uniquely or jointly identified across analyses, with intersections indicated by connected dots below each bar. Genes supported by both transcriptomic and DNA methylation data (dual omics evidence) are annotated. Genes with dual evidence within the primary analysis are shown in bold. (D) Schematic representation of a candidate gene with dual evidence of association with the OIT response, defined as being differentially expressed and either overlapping with or within 5 kb of a DMR, or annotated to a significant DMP. Such loci may also display an eQTM effect. (E–F) Example of an eQTM locus on chromosome 7 involving *TRBV30*: MRNA expression levels of *TRBV30* were significantly correlated with DNA methylation at cg16814513, located ±900 bp downstream, in both unstimulated (E) and peanut‐stimulated (F) PBMCs. Density plots show the distribution of methylation (top) and gene expression (right) in complete (blue) and incomplete (red) responders. Correlation between methylation and expression is displayed (Kendall correlation; unstimulated: *R* = −0.36, *p* < 0.001; stimulated: *R* = −0.21, *p* = 0.007). DEGs, differentially expressed genes; DMPs, differentially methylated probes; DMRs, differentially methylated regions; eQTM, expression quantitative trait methylation; OIT, oral immunotherapy; PBMCs, peripheral blood mononuclear cells; PHA, phytohemagglutinin.

Regression analysis in all PBMC samples identified 184 DEGs significantly associated with the OIT response after correction for multiple testing (Table 1, Table S2). Of these, 46 DEGs were found in unstimulated PBMCs (Figure 3A), while 142 DEGs were specifically detected in peanut‐stimulated PBMCs, but not after non‐specific stimulation with PHA. The detailed analyses (binary or quantitative; stimulated or unstimulated samples) associated with the detection of these 184 genes are summarized in Figure 3A and Table S2.

**TABLE 1:** Differentially expressed genes associated with the binary and quantitative phenotypes.

Gene	Condition^a^	Effect size^b^ (binary/quantitative)	Adjusted *p* ^c^ (binary/quantitative)
*SETBP1*	Medium	−0.090/0.004	3.12 × 10^−5^/2.07 × 10^−2^
*FBN2*	Peanut	0.863/0.482	7.79 × 10^−3^/1.34 × 10^−2^
*MRC2*	Peanut	−0.461/−0.289	9.27 × 10^−4^/2.83 × 10^−4^
*HSPG2*	Peanut	−0.813/−0.347	1.12 × 10^−3^/5.39 × 10^−3^
*IGHE*	Peanut	−1.780/−0.861	1.15 × 10^−3^/1.29 × 10^−3^
*SLC47A1*	Peanut	−1.011/−0.501	2.25 × 10^−3^/4.07 × 10^−3^
*BBS2*	Medium	−0.197/−0.104	4.44 × 10^−3^/1.32 × 10^−2^
*ZC3HAV1L*	Peanut	−0.964/−0.400	3.51 × 10^−3^/2.06 × 10^−2^
*TMEM18*	Medium	−0.154/−0.091	6.95 × 10^−3^/4.61 × 10^−2^
*MAL*	Peanut	−0.609/−0.318	4.94 × 10^−3^/1.98 × 10^−3^
*ATG9B*	Peanut	−0.400/−0.194	7.79 × 10^−3^/3.65 × 10^−3^
*GPR15*	Peanut	0.336/0.223	8.66 × 10^−3^/3.25 × 10^−2^
*C3orf18*	Peanut	−0.233/−0.113	1.80 × 10^−2^/3.08 × 10^−2^
*IL5*	Peanut	−1.134/−0.524	1.98 × 10^−2^/3.32 × 10^−2^
*SBK1*	Peanut	−0.529/−0.236	2.22 × 10^−2^/3.92 × 10^−2^
*NSMCE1*	Peanut	−0.123/−0.096	2.33 × 10^−2^/4.76 × 10^−2^
*SUSD2*	Peanut	−0.366/−0.182	2.41 × 10^−2^/3.44 × 10^−2^
*GYS1*	Peanut	−0.199/−0.098	2.72 × 10^−2^/1.46 × 10^−2^
*MEAK7*	Medium	−0.555/−0.324	4.41 × 10^−2^/1.32 × 10^−2^
*PLPP1*	Peanut	−0.127/−0.064	3.28 × 10^−2^/4.79 × 10^−2^
*FUT7*	Peanut	−0.276/−0.156	3.95 × 10^−2^/3.25 × 10^−2^
*CDIPT*	Peanut	0.012/−0.009	4.16 × 10^−2^/4.79 × 10^−2^
*SERINC5*	Peanut	−0.275/−0.131	4.16 × 10^−2^/6.78 × 10^−3^
*C16orf96*	Peanut	−0.406/−0.237	4.85 × 10^−2^/3.92 × 10^−2^

*Note:* All DEGs associated with both the ‘binary’ (complete Vs. incomplete responders) and ‘quantitative’ (difference in tolerated peanut protein between initial and final OFC) OIT outcomes are displayed. For additional information and the complete list of DEGs, see Table S2.

Abbreviations: DEGs, differentially expressed genes; OFC, oral food challenge; OIT, oral immunotherapy; PBMCs, peripheral blood mononuclear cells.

^a^ ‘Condition’ specifies whether the DEG was detected in stimulated (peanut) or unstimulated (medium) PBMCs.

^b^ Effect sizes are reported as log2fold change. For the ‘binary’ phenotype, a positive effect size indicates higher expression in complete responders, for the ‘quantitative’ phenotype, a correlation with greater improvement during OIT.

^c^ The ‘adjusted *p*‐value’ using the Benjamini‐Hochberg FDR method is shown for both phenotypes.

### 3.3 Functional Enrichment Analysis of DEGs

**FIGURE 4:**
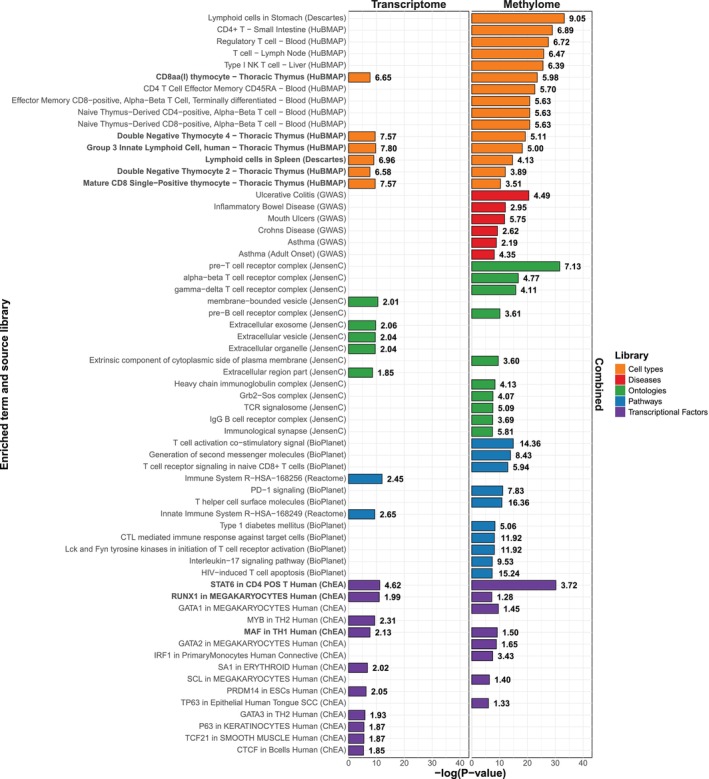
Comparative gene set enrichment analysis for DEGs and genes linked to variation in DNA methylation from combined peanut‐stimulated and unstimulated PBMCs. This figure displays the top 10 gene sets based on odds ratio, plus any additional terms overlapping across RNA and DNA methylation data sets, identified from two distinct data sets: 184 DEGs (left) and 1001 genes annotated to DNA methylation differences (right), from combined peanut‐stimulated and unstimulated PBMCs. Terms are grouped into functional domains: Cell Types, Ontologies, Pathways, Transcription Factors, and Diseases. The library from which each enriched term was identified is shown in parentheses. Gene set enrichment significance is indicated by –log_10_ *p*‐values. Odds ratios are shown in bold at the end of each bar. Complete enrichment results are provided in Tables S3 and S25.

To understand the biological role of transcriptional changes associated with the OIT response, we performed functional enrichment analyses using the Enrichr platform for the 184 DEGs (Figure 4). Significant genes defining the enrichment are listed in Table S3. Notably, the most enriched cell type was group 3 innate lymphoid cells (ILC3). Significant enrichment was also found in in early T cell development stages such as double negative and CD8 single‐positive thymocytes, and the CD8αα subset of CD8^+^ T cells. Additionally, DEGs pointed to exosomes as a significantly enriched subcellular compartment. Exosomal genes associated with complete desensitization revealed a strong enrichment of gastrointestinal tissue (Figure S8). Furthermore, we aimed to identify transcription factors associated with the gene expression changes. The strongest enrichment was found for STAT6 in CD4+ T cells, a key inducer and regulator of Th2 responses. Additional enriched transcription factors included RUNX1 in megakaryocytes, MYB in T‐helper 2 cells and MAF in T‐helper 1 cells (Figure 4). Performing the enrichment analysis separately for 142 DEGs from peanut‐stimulated and 46 DEGs from unstimulated PBMCs demonstrated that these functional annotations originated from the peanut‐specific regulatory changes, while there was minimal overlap of enrichment terms found in unstimulated PBMCs (Figure S9, Table S3).

### 3.4 Subgroup‐Specific Gene Expression Analysis

Additional DEGs were identified in the subgroup analyses pre−/post OIT and within complete and incomplete responders (Figure 1E, Table S4). Notably, 11 DEGs (*CCDC6, ARRDC4, RNF125, ATP2C1, SELENOI, MCL1, BCL11B, SFT2D2, VIRMA, GCNT2, IDE*) were found in samples collected exclusively before OIT (Table S5, Figure S10), warranting further investigation to explore their predictive utility. Detailed subgroup results are shown in Tables S4–S8.

WGCNA revealed 4 significant gene networks (modules) of highly interconnected genes of which 2 were found in each the complete and incomplete responders (Figure S11, Table S9). In the complete responders the *Firebrick* module of 1921 genes revealed an enrichment for intestinal tuft cells, regulatory T cells (Tregs), and memory T/B cells, with pathways implicating B cell receptor inhibition and base excision repair which plays a key role in immunoglobulin class switch recombination and somatic hypermutation (Figure S12, Table S10). The enrichment of the second complete responder module (*darkgrey*, 1541 genes) included cytotoxic/memory T cells, Th17 and γδ T cells alongside robust enrichment for ribosomal biogenesis/translation (Figure S13, Table S11). In contrast, two gene networks identified in incomplete responders emphasized myeloid cells (monocytes, dendritic cells, neutrophils) and innate immune processes (*gainsboro* module of 68 genes, Figure S14, Table S12) as well as hypoxia adaptation (HIF‐1/HIF‐2 signaling), glycolysis (SLC2A1/3), and angiogenesis (VEGF‐A), regulated by RELB (NF‐κB) and SMAD3 (*lightcoral* module of 186 genes, Figure S15, Table S13).

### 3.5 Peanut Desensitization Is Associated With Differential DNA Methylation of 1001 Genes

Like the transcriptome analysis, DNA methylation changes were analyzed using the binary or quantitative OIT outcomes in peanut‐stimulated and unstimulated PBMCs separately. DNA methylation analysis was conducted on two levels: first, testing for methylation changes at individual cytosine‐phosphate‐guanine sites (CpG), identifying 55 differentially methylated probes (DMPs, Table 2, Table S14). Secondly, as DNA methylation typically occurs across multiple CpGs within a regulatory region, we analyzed the methylation status of adjacent DMPs to identify differentially methylated regions (DMRs). After correction for multiple testing, 875 unique DMRs were associated with a stronger OIT response (Table 2, Table S15). Simulated epigenome‐wide association analyses confirmed the robustness of the results (Figure S2). Significant DMPs and DMRs were annotated to 1001 genes, with 425 detected in unstimulated and 903 in peanut‐stimulated PBMCs (Figure 3B). This difference underscores the importance of analyzing DNA methylation and gene expression changes under allergen‐specific stimulation conditions.

**TABLE 2:** Top 10 differentially methylated probes (DMPs) and top 10 differentially methylated regions (DMRs) associated with outcomes of oral immunotherapy.

**DMPs from unstimulated PBMCs**											
**Binary outcome**						Quantitative outcome					
**DMwP** ^**a**^	**Chr**	**Position**	**Coefficient** ^**b**^	***p***	**Gene** ^**c**^	**DMP** ^**a**^	**Chr**	**Position**	**Coefficient** ^b^	***p***	**Gene** ^**c**^
**cg10983013**	**6**	**170816044**	**−0.2129**	**2.15 × 10** ^**−10**^	**—**	**cg10983013**	**6**	170816044	**0.1546**	**5.52 × 10** ^**−10**^	***—***
**cg00555811**	**2**	**46523319**	**0.0527**	**2.14 × 10** ^**−9**^	***EPAS1***	**cg02525719**	**4**	183728549	**−0.0224**	**9.09 × 10** ^**−10**^	***—***
**cg02525719**	**4**	**183728549**	**0.0296**	**3.12 × 10** ^**−9**^	***—***	**cg02306229**	**4**	1557317	**−0.0162**	**9.08 × 10** ^**−9**^	***—***
cg03058874	19	34801449	0.0916	3.88 × 10^−9^	*GARRE1*	**cg16814513**	**7**	142511983	**0.0412**	**1.07 × 10** ^**−8**^	***—***
**cg16814513**	**7**	**142511983**	**−0.056**	**8.95 × 10** ^**−9**^	***—***	cg19066078	12	130571342	0.0226	1.15 × 10^−8^	*—*
**cg02306229**	**4**	**1557317**	**0.0212**	**4.21 × 10** ^**−8**^	***—***	**cg00555811**	**2**	46523319	**−0.0375**	**1.16 × 10** ^**−8**^	***EPAS1***
cg00644629	1	29598263	−0.0417	6.84 × 10^−8^	*PTPRU*	cg20474450	14	56777463	−0.0305	1.45 × 10^−8^	*—*
cg17518825	2	46523461	0.0751	7.28 × 10^−8^	*EPAS1*	cg09197432	4	183729176	−0.0441	3.02 × 10^−8^	*—*
cg05154177	5	133120839	−0.0171	8.10 × 10^−8^	—	cg16950831	1	40618846	0.0516	3.51 × 10^−8^	*—*
cg09345949	14	106709227	0.1047	8.26 × 10^−8^	—	cg22283921	8	95961819	0.0306	3.82 × 10^−8^	*TP53INP1*
**DMRs from unstimulated PBMCs**											
**Chr**	**DMR start**	**DMR size**	**Number of CpGs**	***p***_**FDR**_ ^**e**^		**Phenotype**		**Genes** ^**d**^			
10	14008305	1724	14	4.04 × 10^−19/^3.67 × 10^−18^		Binary/quantitative		ENSG00000229751*/**FRMD4A***			
8	94949590	645	9	4.04 × 10^−19/^1.28 × 10^−17^		Binary/quantitative		***NDUFAF6/*ENSG00000253878*/*** *TP53INP1*			
16	1533389	1126	13	5.71 × 10^−19^		Quantitative		***TMEM204/IFT140/*** ENSG00000280062			
7	182807325	1620	5	1.33 × 10^−18^		Quantitative		*TRBV30*			
3	46717605	603	9	1.78 × 10^−18^		Binary		**ENSG00000206549*/PRSS50/*** *PRSS46P*			
4	182807325	983	6	2.07 × 10^−17/^6.71 × 10^−18^		Binary/quantitative		*TENM3*			
13	36297508	701	12	6.73 × 10^−16^		Binary		***CCDC169‐SOHLH2/CCDC169/**SPART*			
13	87675753	1672	10	1.08 × 10^−14^		Binary		***SLITRK5/**MIR4500HG*			
11	76656880	770	5	2.55 × 10^−14^		Quantitative		***LRRC32/*ENSG00000236304*/*ENSG00000236304**			
7	142796463	345	7	3.07 × 10^−14^		Quantitative		*TRBC1/TRBJ2‐1/**TRBJ2‐2/TRBJ2‐2P**/TRBJ2‐3/TRBJ2‐4/TRBJ2‐5/TRBJ2‐6/TRBJ2‐7/TRBC2/*ENSG00000289416			
**DMPs from peanut‐antigen stimulated PBMCs**											
**Bzinary outcome**						**Quantitative outcome**					
**DMP** ^**a**^	**Chr**	**Position**	**Coefficient** ^**b**^	***p***	**Gene** ^**c**^	**DMP** ^a^	**Chr**	**Position**	**Coefficient** ^b^	***p***	**Gene** ^**c**^
**cg16814513**	**7**	**142511983**	**−0.0726**	**1.03 × 10** ^**−10**^	**—**	**cg05081118**	**22**	**22299432**	**−0.0346**	**8.82 × 10** ^**−12**^	***PPM1F***
**cg09002358**	**5**	**57541568**	**0.0673**	**1.27 × 10** ^**−10**^	**—**	**cg09002358**	**5**	**57541568**	**−0.0513**	**5.84 × 10** ^**−11**^	***—***
**cg10983013**	**6**	**170816044**	**−0.2092**	**1.56 × 10** ^**−10**^	**—**	**cg10983013**	**6**	**170816044**	**0.1558**	**2.84 × 10** ^**−10**^	***—***
**cg00555811**	**2**	**46523319**	**0.0519**	**2.65 × 10** ^**−10**^	***EPAS1***	**cg17908151**	**7**	**92646595**	**−0.0463**	**3.29 × 10** ^**−10**^	***—***
**cg26742187**	**16**	**1131907**	**−0.0359**	**1.47 × 10** ^**−9**^	***—***	**cg16814513**	**7**	**142511983**	**0.0528**	**6.50 × 10** ^**−10**^	***—***
cg12701013	1	160678921	−0.0735	1.77 × 10^−9^	*CD48*	**cg00555811**	**2**	**46523319**	**−0.0379**	**1.37 × 10** ^**−9**^	***EPAS1***
**cg05081118**	**22**	**22299432**	**0.0409**	**5.77 × 10** ^**−9**^	***PPM1F***	cg06482188	17	77644545	0.0356	2.55 × 10^−9^	*—*
cg00817731	3	46759475	−0.0676	2.46 × 10^−8^	*PRSS50*	cg14597964	11	62193135	−0.0065	3.00 × 10^−9^	*—*
cg20739426	1	202659201	−0.0311	2.81 × 10^−8^	*SYT2*	**cg26742187**	**16**	**1131907**	**0.0265**	**3.71 × 10** ^**−9**^	***—***
cg23523711	2	392873	−0.0327	3.73 × 10^−8^	**—**	cg21371809	10	14052028	0.0401	6.04 × 10^−9^	*FRMD4A*
**DMRs from peanut‐antigen stimulated PBMCs**											
**Chr**	**DMR start**	**DMR size**	**Number of CpGs**	***p***_**FDR**_ ^**e**^		**Phenotype**		**Genes** ^**d**^			
10	14008305	1724	14	4.04 × 10^−19/^3.67 × 10^−18^		Binary/quantitative		ENSG00000229751*/**FRMD4A***			
8	94949590	645	9	4.04 × 10^−19/^1.28 × 10^−17^		Binary/quantitative		***NDUFAF6/*ENSG00000253878*/*** *TP53INP1*			
16	1533389	1126	13	5.71 × 10^−19^		Quantitative		***TMEM204/IFT140/*** ENSG00000280062			
7	142813594	1620	5	1.33 × 10^−18^		Quantitative		*TRBV30*			
3	46717605	603	9	1.78 × 10^−18^		Binary		**ENSG00000206549*/PRSS50/*** *PRSS46P*			
4	182807325	983	6	2.07 × 10^−17/^6.71 × 10^−18^		Binary/quantitative		*TENM3*			
13	36297508	701	12	6.73 × 10^−16^		Binary		***CCDC169‐SOHLH2/CCDC169/**SPART*			
13	87675753	1672	10	1.08 × 10^−14^		Binary		***SLITRK5/**MIR4500HG*			
11	76656880	770	5	2.55 × 10^−14^		Quantitative		***LRRC32/*ENSG00000236304*/*ENSG00000236304**			
7	142796463	345	7	3.07 × 10^−14^		Quantitative		*TRBC1/TRBJ2‐1/**TRBJ2‐2/TRBJ2‐2P**/TRBJ2‐3/TRBJ2‐4/TRBJ2‐5/TRBJ2‐6/TRBJ2‐7/TRBC2/*ENSG00000289416			

*Note:* This table displays the top 10 DMPs and the top DMRs associated with OIT outcomes in unstimulated (top half) and peanut‐stimulated (bottom half) PBMCs. DMPs are shown for the binary (left) or quantitative (right) phenotype. Detailed information and the complete list of DMPs can be found in Table S14. For the top DMRs, the chromosome, starting position (DMR st245:378art), size in base pairs (DMR size) and number of CpGs defining each region are shown. Further details and expanded lists of DMRs can be found in Table S15.

Abbreviations: chr, chromosome; CpG, cytosine‐phosphate‐guanine site; DMPs, differentially methylated probes; DMRs, differentially methylated regions; OIT, oral immunotherapy; PBMCs, peripheral blood mononuclear cells.

^a^ Significant DMPs identified in more than one analysis are highlighted in bold.

^b^ DMPs with a positive coefficient indicate higher methylation levels in incomplete responders (binary phenotype) and in those showing less improvement during OIT (quantitative phenotype).

^c^ Gene indicates the Illumina annotated reference gene.

^d^ Genes within 5 kb from the DMR are shown. For genes without an assigned gene symbol, the Ensembl identifier is displayed. Genes overlapping the DMR are highlighted in bold.

^e^ *p*‐value_FDR_ indicates the false‐discovery rate adjusted *p‐*value for the binary/quantitative OIT outcome.

Methylation subgroup analyses (Table S3) only identified 2 DMPs (linked to GPR113; SELENOI and SORCS1) in PBMC samples after OIT (Table S16), while no individual DMP was found in PBMC samples obtained before OIT, within the complete or incomplete responders (Table S17–S19). However, several DMRs were found for the subgroup analysis after collapsing individual CpGs, except intra‐complete responders (Table S20–S22) including 48 DMRs as pre‐treatment markers of stronger OIT outcomes (Table S20). For 16 of their 48 annotated protein‐coding genes, the gastrointestinal tract was among the top 3 sites of expression in the GTEx database (Table S23) [23].

### 3.6 Dual Epigenetic and Transcriptomic Association With OIT Outcome

Differential DNA methylation observed in response to OIT is thought to influence treatment outcome by regulating gene expression. We identified 16 genes showing associations in both transcriptome and methylome in the primary analysis (Figure 3C). A hypergeometric test confirmed the significance of this overlap (*p‐*value = 0.017). In the subgroup analyses, 7 additional DEGs with dual epigenetic and transcriptomic associations were identified (Figure 3C), including *GCNT2* as the only gene detected in samples exclusively collected before OIT. To further explore the relationship between DNA methylation and gene expression, we investigated whether methylation levels correlated with gene expression levels (eQTMs). We identified 11 DMPs paired with 11 DEGs within a 1.5 Mb region. After adjusting for false‐discovery rate, we detected 5 DMPs with significant eQTM effect on 4 DEGs (*TRBV30*, *CD22*, *TBL3*, *WBP2*, Figure 3D–F, Table S24), suggesting epigenetic modulation of gene expression relevant to OIT efficacy.

### 3.7 Enrichment Analysis of OIT‐Associated DNA Methylation Changes

We performed functional enrichment analyses on genes linked to alterations in DNA methylation (DMPs, DMRs, and eQTMs) for 425 in unstimulated and 903 in peanut‐stimulated PBMCs separately (Figure S9, Table S25), as well as for 1001 unique genes in combination (Figure 4). When we look at 1001 unique genes, 146 immune cell types were enriched (Table S25).

The top‐ranked cell types were lymphoid cells in the stomach, CD4+ T cells in the small intestine, and regulatory T (Treg) cells in blood. There was significant overlap with the immune cell types identified through transcriptome analysis (hypergeometric test, *p‐*value = 0.018). All 6 significantly enriched immune cell types detected through transcriptome analysis were also identified through differential DNA methylation, including gut‐resident innate (ILC3) and adaptive immune cells (the CD8αα subset of CD8+ T cells). Regarding human disease associations, the top 4 of 6 enriched terms involved inflammation in the gastrointestinal tract, emphasizing gut immune processes in the OIT response. The top three subcellular compartments were T cell receptor complexes, including the gamma‐delta T cell receptor complex, found on intraepithelial lymphocytes that are most abundant in the gut. Since DNA methylation can modulate the binding of transcription factors to DNA, we also performed a transcription factor enrichment analysis. Similar to the enrichment of DEGs, STAT6 in CD4+ T cells was the most significant transcription factor. Moreover, RUNX1 in megakaryocytes and MAF in T‐helper 1 cells were also significantly enriched in both analyses (Figure 4, Table S25). Using permutation studies, we demonstrated that the sharing of functional annotations detected in 2 different experimental approaches (mRNA sequencing and DNA methylation profiling) is highly significant (empirical *p*‐value < 0.001).

### 3.8 Comparison of Active Treatment Versus Placebo in Peanut‐Allergic Children Undergoing OIT

Comparing active treatment to placebo after intervention revealed immunoglobulin genes (*IGH, IGK, IGL*) dominating the top 50 DEGs in both unstimulated (45/50 genes) and peanut‐stimulated (21/50 genes) PBMCs. All immunoglobulin genes were downregulated in the active treatment group. In contrast, there were no significant differences after PHA stimulation. After multiple‐testing adjustment, five immunoglobulin genes were significantly downregulated in the active group (unstimulated: *IGKV6‐21, IGKV1‐27, IGKV1‐6*; peanut‐stimulated: *IGKV2‐30, IGKV1‐6, IGHV3‐*53; Table S26). Baseline comparisons showed minimal pre‐OIT differences (2 DEGs: *CXCL1, PLEKHH2*; one immunoglobulin gene in the top 50; Table S27). Similarly, methylation analysis identified no DMPs (Tables S28 and S29) and only 4 and 2 DMRs before (Table S30) and after OIT (Table S31).

## 4 Discussion

Defining factors mediating the variability in OIT responses is a major challenge in precision medicine for peanut allergy. Without established biomarkers to predict OIT efficacy, we aimed to identify molecular changes distinguishing complete from incomplete desensitization responses. Using multi‐omics profiling of PBMCs, we identified differential gene expression and DNA methylation patterns highlighting immune processes in the gut as critical components of the OIT response. A major strength of our study is the use of standardized OFCs to capture variations in treatment outcome precisely. Furthermore, the inclusion of placebo‐treated individuals, the longitudinal investigation of each participant before and after OIT, and of each PBMC sample with and without allergen‐specific stimulation effectively increased study power. Most transcriptomic associations were detected after peanut stimulation. By performing non‐specific stimulation with PHA, we demonstrate that those changes were antigen‐specific.

Multiple studies reported a decrease in peanut‐specific [24, 25, 26, 27, 28, 29, 30] or peanut‐component‐specific IgE after OIT [24, 25, 26]. We found significantly higher IgE levels of peanut‐ and Ara h 2‐specific IgE in incomplete compared to complete responders, with a slight but insignificant decrease after treatment within each group. Congruently, the initial eliciting and maintenance dose were lower in the incomplete responders, reflecting a heightened Th2 state associated with a weaker OIT response. This is consistent with studies where suppression of Th2/Th2A signatures resulted in more positive OIT outcomes [12, 31]. Regarding IgG4, our findings align with previous research showing increased levels after OIT [6, 24, 25, 27, 28, 32, 33, 34], but contrary to expectations, this increase was driven by incomplete responders, questioning its utility as a marker for OIT success. It remains to be explored if the upregulation of peanut‐specific IgG4 reflects a distinct regulatory mechanism in this group of patients. Alternatively, it has been shown that the epitope specificity of the neutralizing antibodies rather than their levels correlated with the clinical efficacy of OIT [35]. Regarding the IgG4 to IgE ratio, the IMPACT trial reported an increase during OIT which was more pronounced in peanut allergic patients who achieved desensitization. Significance was only reported for the remission group after 134 weeks of OIT plus 26 weeks of avoidance [24]. In our OIT trial, we observed the same trend of a higher IgG4 to IgE ratio in complete responders which, however, did not reach significance likely due to a shorter duration (66 Vs. 160 weeks) and different outcome definitions.

Unlike humoral markers, Th2 cytokine secretion remains less explored in OIT outcomes. Previous studies showed a decrease in PBMC‐secreted IL‐4, IL‐5, IL‐13, and IL‐9 after OIT [9, 33, 36]. We demonstrate that the decrease in Th2 cytokine secretion was primarily driven by incomplete responders. They exhibited significantly higher levels of IL‐4 and IL‐5 before OIT, which later declined to levels approaching those initially observed in complete responders—consistent with findings from the IMPACT study, where effective OIT shifted Th2A‐high profiles toward a Th2A‐low immunotype. This pattern suggests that patients with higher baseline production of IL‐4 and IL‐5 may require longer treatment to achieve complete desensitization. Alternatively, medication‐induced reduction of Th2 activation before or during OIT may be a promising approach to improve treatment success. In recent peanut OIT trials, co‐treatment with omalizumab (anti‐IgE) or dupilumab (anti‐IL‐4Rα) had a positive effect on the outcome [37, 38].

Overall, we observed many more changes in DNA methylation than RNA expression associated with a stronger OIT response. This may be due to methylation changes detected for genes not expressed in PBMCs. Recent studies have shown that the genetic regulation of DNA methylation observed in blood cells occurs concordantly at 72%–86% of DNA methylation sites in distant tissues, such as adipose tissue [39]. This demonstrates that methylome analysis in blood cells enables insights into methylation changes relevant in other tissues [39]. In our study, for example, hypomethylation at the envoplakin gene (*EVPL*), which is not expressed in blood but most abundantly in esophagus and skin [23], was associated with a better OIT response. EVPL forms heterodimers with periplakin and acts as a barrier protein linking desmosomes and intermediate filaments to the cornified envelope [40]. Intriguingly, mutations in periplakin cause eosinophilic esophagitis [41], which is often associated with peanut allergy [42]. Our finding may suggest that epigenetic regulation of esophageal barrier function is important in the OIT response. However, esophageal biopsies were not available in our study, and we did not demonstrate concordant DNA methylation patterns between PBMCs and the esophagus. Thus, additional studies are needed to resolve the functional impact of EVPL hypomethylation.

To better understand patterns of gene expression, we further investigated the transcriptomic changes during OIT in the complete and incomplete responders separately. WGCNA revealed distinct molecular signatures in each outcome group. Complete responders exhibited modules balancing specific immune responses (Treg, B cell inhibition), effector adaptation (cytotoxic T cells, ribosomal translation), and involvement of the intestinal milieu (intestinal tuft cells, γδ T cells). These findings align with previous reports of Treg expansion and Th2 gene signature suppression in successful OIT [11, 31]. In contrast, incomplete responders displayed metabolic stress (hypoxia, glycolysis) and myeloid‐driven inflammation (NLRP1, HIF‐1 signaling pathway). Interestingly, HIF‐1, a key regulator of the hypoxia response, plays an important role in innate immune responses in mucosal inflammation [43], including eosinophilic esophagitis [44]. Taken together, our findings suggest a critical imbalance of robust Treg responses and B cell suppression on the one hand and the dominance of innate immune signaling and metabolic stress on the other as potential mechanism of incomplete desensitization.

We found a marked downregulation of immunoglobulin genes in patients receiving peanut compared to placebo. A study of the peanut‐specific antibody repertoire of peanut‐allergic, peanut‐sensitized, but tolerant and non‐atopic individuals has shown that peanut allergy is associated with dominant usage of the variable heavy gene 3 (V_H_3) family, especially IGHV3‐30, VH3‐23 and VH3‐72 [45]. Notably, 10 of 13 V_H_3 transcripts, including IGHV3‐30, VH3‐23, and VH3‐72, were significantly downregulated after peanut treatment in our study and overlapped with peanut allergic individuals studied by Ehlers et al. However, additional experimental approaches are required to understand the link of these expression changes to antibody specificity and class. We performed separate gene set enrichment analyses for genes with significant changes in RNA expression or DNA methylation to understand their role in the treatment response. There was an intriguing overlap between both analyses with several enriched domains pointing to immune processes in the gut. All 6 immune cell types enriched for DEGs were also detected through DNA methylation changes.

Group 3 innate lymphoid cells (ILC3) are most abundant in the gut where they regulate intestinal barrier function and suppress inflammatory responses [46]. In blood, circulating ILC3 (cILC3) precursors exist whose frequency is age‐dependent with highest levels in childhood [47]. They are specifically dysregulated in several inflammatory diseases such as Crohn's disease and asthma [48]. In a mouse model, ILC3s were involved in tolerance to dietary antigens through the induction of intestinal Treg cells [49]. Moreover, antigen‐presenting ILC3s limited allergen‐specific T cells and airway inflammation in mice suggesting a regulatory role in the adaptive immune response [50]. Other enriched cell types were early T‐cell developmental stages and the CD8αα subset of CD8+ T cells in the thymus. CD8αα intraepithelial lymphocytes (IELs) home to the gut where they play an important role in intestinal immunity [51]. Notably, CD8αα‐positive IELs in the gut have the highest abundance of the γδ T‐cell receptor, a subcellular compartment prominently enriched in the differential methylation analysis and in the co‐expression network of the complete responders. γδ T cells which develop in the thymus make up to 16% of circulating T cells and migrate to the gut [52]. In a mouse model, the induction of peanut sensitization was associated with a significant reduction in intestinal γδ T cells [53]. Blocking the γδ T cell receptor amplified allergic responses characterized by peanut‐specific IgE and Th2 cytokine production in splenocytes. A recent study demonstrated that intestinal γδ T cells modulate tissue responses to dietary nutrients by suppressing IL‐22 production from ILC3s, establishing a link between γδ T cells, ILC3s, and intestinal adaptation to nutritional exposures [54]. The enrichment of DEGs emphasized a role for extracellular exosomes in peanut desensitization. Exosomes are small vesicles crucial for transporting and processing proteins, nucleic acids and metabolites. Network analysis of the exosomal genes associated with a stronger OIT response highlighted the gastrointestinal tract as relevant tissue. They exert different immunological functions, including antigen presentation, Th2 proliferation, and differentiation [55, 56]. Indeed, they have been called “tolerosomes” for their role in mediating oral tolerance development. In a mouse model, oral administration of OVA resulted in the generation of MHC class II vesicles by intestinal epithelial cells that could be transferred to naïve litter mates to suppress OVA‐specific immune responses [57]. Thus, exosomal antigen processing may influence the magnitude of treatment response and offer a new target for treatment optimization.

Both transcriptome and DNA methylome profiling highlighted three transcription factors: STAT6 in CD4+ T cells, RUNX1 in megakaryocytes, and MAF in T‐helper 1 cells. All three transcription factors were also detected in the subanalyses of peanut‐stimulated PBMCs and in the complete responders, underlining their potential role in stronger OIT responses. STAT6 plays a crucial role in allergic phenotypes. Rare gain‐of‐function mutations in STAT6 cause severe early‐onset allergic diseases, including food allergy [58]. Due to both activating and repressive effects of STAT6 on gene expression [59, 60], we cannot make a claim on the effect direction in our study. While one would expect STAT6 repression to underlie the complete response to OIT, this remains to be shown in future studies. Similarly, MAF (or c‐MAF) has recently been shown to suppress intestinal inflammation [61]. It is preferentially expressed in intestinal ILC3s, acting as a key regulator of ILC3 identity and functional plasticity [62]. Finally, RUNX1 is a hematopoietic transcription factor involved in platelet production [63] and T cell differentiation, but its precise role in tolerance development remains to be elucidated.

We performed a separate analysis focusing only on pre‐OIT samples to find potential predictive markers for the OIT response. We detected 11 significant DEGs, including *GCNT2*, which was also identified by changes in DNA methylation. Methylation change within a regulatory region upstream of *GCNT2* has also been identified as mediating tolerance in subcutaneous immunotherapy for birch pollen allergy [64]. While the role of most of these genes in peanut allergy has not been investigated, overexpression of myeloid cell leukemia‐1 (MCL‐1) protects eosinophils from apoptosis and exacerbates allergic inflammation [65]. In our study, lower expression of *MCL1* was associated with a better OIT response, suggesting reduced MCL‐1 activity before OIT may improve treatment efficacy. Furthermore, arrestin domain containing 4 (*ARRDC4*) is highly expressed in neuronal tissues, the colon, and esophagus. It regulates exosome biogenesis and protein transport, again emphasizing gastrointestinal exosomal allergen processing in the peanut OIT response [66].

Genes with methylation alterations before treatment were prominently expressed in the gastrointestinal tract (Table S23). For example, NLR family pyrin domain containing 6 (*NLRP6*) is predominantly expressed in the small intestine and has been shown to suppress Th2 responses, reinforcing its role in OIT success [67]. Moreover, PCDC1 (or PD‐1) is a receptor on activated T‐cells that plays a critical role in the induction and maintenance of peripheral tolerance. PD‐1 blockade inhibited anaphylaxis in a mouse model of peanut allergy [68]. Our enrichment analysis of methylation changes highlighted the significance of PD‐1 signaling (Figure 4, Table S25), suggesting that epigenetic regulation of other genes in this pathway may also impact the strength of the OIT response. Thus, we present a catalog of DEGs and DMRs detected before treatment that should be explored further for their predictive potential in independent data sets.

Our study has certain limitations. Due to the extensive clinical and molecular phenotyping of trial patients, the sample size was limited. While we report associations after rigorous statistical analysis and multiple testing corrections, independent replication was not possible due to the lack of comparable data sets. Nonetheless, the significant overlap among identified genes, cell types, and transcription factors detected by transcriptomic and epigenomic analysis supports the validity of our results. Selecting the best timing and conditions for molecular profiling and defining OIT outcome remains challenging. We used 4500 mg and ≤ 1000 mg of peanut protein to define complete and incomplete responders. Despite the lack of standardized thresholds, 1000 mg of peanut protein has been suggested as a clinically meaningful threshold to reduce the risk of inadvertent allergic reactions in everyday life [69], and tolerance of similar high doses (4000 [8] and 5000 mg [24, 70]) have been used to assess OIT efficacy in other studies. By additionally using the increase in tolerated dose as a quantitative phenotype, we aimed to capture a broader range of potential outcomes. Finally, we focused on PBMCs as multiple cell types are thought to play a role in tolerance induction [71] and due to their accessibility for repeated sampling in pediatric populations. On the contrary, studying heterogeneous PBMCs may have missed signals from specific immune cell subsets. Although the results of our study pointed to an involvement of intestinal immunity in the OIT response, the respective tissue was not available for validation. A high correlation of DNA methylation between blood and adipose tissue has been demonstrated [39], but for gastrointestinal tissues data are lacking. However, we identified genes and pathways involved in intestinal immunity in several independent analyses thus providing a valuable resource for future investigations.

In summary, we present a detailed catalog of differentially expressed and/or methylated genes associated with the magnitude of the peanut OIT response. Our findings emphasized gastrointestinal immune processes in OIT responsiveness, highlighting specific innate and adaptive immune cells, alongside exosomal allergen processing, as target for future investigations. The divergence between regulatory T‐cell dominance and B cell suppression in complete responders and unresolved innate signaling in incomplete responders identifies pathways for potential therapeutic intervention. These findings lay the foundation for developing predictive biomarkers and personalized treatment strategies in peanut allergy.

## Author Contributions

Conceptualization: K.Bl., I.M., N.H., K.Be., A.A.‐S., Y.‐A.L. Methodology: A.A.‐S., S.E.A., A.G., A.C.S.N.J., I.M., K.Bl., P.C., A.I., N.H., K.Be., Y.‐A.L. Patient recruitment, clinical investigation, and biobanking: K.Bl., K.Be. Experimental analysis: S.E.A., A.G., A.C.S.N.J., I.M., K.Bl., P.C., A.I. Data analysis/interpretation: A.A.‐S., A.G., A.C.S.N.J., I.M., N.H., Y.‐A.L. Supervision: K.Be., Y.‐A.L. Writing – original draft: A.A.‐S., I.M., Y.‐A.L. Writing – review and editing: All authors.

## Conflicts of Interest

KBe reports advisory board/consulting fees or speaker's bureau fees from Aimmune Therapeutics, Bencard, Danone/Nutricia, DBV, Hycor, Infectopharm, Mabylon, Meda Pharma/Mylan, Nestle, Novartis, and ThermoFisher; and research grants from Aimmune, ALK, Danone/Nutricia, DBV Technologies, Hipp, Hycor, Infectopharm, and Novartis outside the submitted work. KBl or her institution received grants from Aimmune therapeutics, DBV Technologies, Novartis Pharma GmbH, Hipp GmbH, consulting fees from Aimmune therapeutics, DBV Technologies, Novartis Pharma GmbH, Bencard Allergie, honoraria for lectures from Aimmune therapeutics, DBV Technologies, Novartis Pharma GmbH, HAL Allergy, Allergopharma, Nestle, Thermo Fisher Scientific, Viatris Healthcare GmbH, payment for expert testimony from Nestle, support for travel/meetings from Aimmune therapeutics, DBV Technologies, Bencard Allergie, and ALK. She is on the safety board of an investigator‐initiated study on peanut allergy at Charité, Berlin, and is a board member of the Westgerman Society of Pediatric Allergology. The other authors declare no conflicts of interest.

## Supporting information


Table S1–S31

Appendix S1

## Data Availability

The data that supports the findings of this study are available in the Supporting Information of this article.
