# Supplementary material for: Understanding the Variability of Peanut‐Oral Immunotherapy Responses by Multi‐Omics Profiling of Immune Cells
Source: Allergy. 2025 Jul 22;80(12):3342–58. doi: 10.1111/all.16627 (PMC12666752; doi:10.1111/all.16627)
Supplement: Supplementary file 2 — Appendix S1 [file ALL-80-3342-s002.docx]

##### Supplementary Information

##### Understanding the variability of peanut-oral immunotherapy responses by multi-omics profiling of immune cells

Aleix Arnau-Soler,^1,2,3^ [0000-0001-9768-0513]; Sarah E. Ashley,^1,2,^**^†^** [0000-0001-9973-9742]_,_ Ahla Ghauri,^1,2^ [0000-0003-4665-3624]; Alexander C. S. N. Jeanrenaud,^1,2^ [0000-0002-4230-9976]; Ingo Marenholz,^1,2^ [0009-0007-6933-3849]; Katharina Blumchen,^4^ [0000-0001-5129-852X]; Penelope Cibin,^1,2^ [0009-0008-7582-9653]; Alisa Iakupova,^1,2^ [0000-0002-9452-4010]; Norbert Hubner,^1^ [0000-0002-1218-6223]; Kirsten Beyer,^3,5,‡^ [0000-0003-1859-0419];
Young-Ae Lee^1,2,3,‡^ [0000-0002-1817-9163]

**Affiliations:**

1 Max-Delbrück-Center for Molecular Medicine, Berlin, Germany

2 Clinic for Pediatric Allergy, Experimental and Clinical Research Center of Max-Delbrück-Center for Molecular Medicine and Charité-Universitätsmedizin Berlin, Berlin, Germany

3 German Center for Child and Adolescent Health (DZKJ), partner site Berlin, Berlin, Germany

4 Department of Pediatrics, Division of Pneumology, Allergology, Infectious Diseases and Gastroenterology, Goethe University Frankfurt, Frankfurt am Main, Germany

5 Department of Pediatric Respiratory Medicine, Immunology, and Intensive Care Medicine, Charité-Universitätsmedizin Berlin, Berlin, Germany

**^†^** Present address: Murdoch Children's Research Institute, Parkville, VIC, Australia

‡ These authors codirected the study.

[1. Experimental methods 4](#_Toc194674758)

[1.1. Isolation of peripheral blood mononuclear cells (PBMCs) 4](#_Toc194674759)

[1.2. Stimulation of PBMCs 4](#_Toc194674760)

[1.3. Peanut extract preparation for stimulation of PBMCs 4](#_Toc194674761)

[1.4. Cytokine levels in cell culture supernatants 5](#_Toc194674762)

[1.5. DNA and RNA isolation 5](#_Toc194674763)

[1.6. Bulk RNA sequencing and methylation array 5](#_Toc194674764)

[2. Statistical analysis 6](#_Toc194674765)

[2.1. Deconvolution of PBMC composition 6](#_Toc194674766)

[2.2. Generation of in-house PBMC scRNA-seq datasets 7](#_Toc194674767)

[2.2.1. PBMCs isolation and stimulation 7](#_Toc194674768)

[2.2.2. PBMC processing on the 10X Genomics platform and sequencing 7](#_Toc194674769)

[2.2.3. Data processing and downstream analysis 7](#_Toc194674770)

[2.3. Gene expression analysis 7](#_Toc194674771)

[2.3.1. Primary analysis: Overall effects on desensitisation 8](#_Toc194674772)

[2.3.2. Subgroup analyses and specific models 9](#_Toc194674773)

[1. Pre-OIT samples: Predictive biomarkers in verum participants. 9](#_Toc194674774)

[2.4. Weighted Gene Co-expression Network Analysis (WGCNA) 10](#_Toc194674775)

[2.5. Epigenome-wide association analysis of CpGs and differentially methylated regions (DMRs) 11](#_Toc194674776)

[2.5.1. Subgroup analyses and specific models 12](#_Toc194674777)

[2.5.2. Integration of gene expression with DNA methylation changes 12](#_Toc194674778)

[2.5.3. Functional enrichment analysis 12](#_Toc194674779)

[3. Supplementary References 14](#_Toc194674780)

[4. Supplementary Figures 16](#_Toc194674781)

[Figure S1. Increments in tolerated peanut protein after OIT. 16](#_Toc194674782)

[Figure S2. Histogram of simulated epigenome-wide analysis. 17](#_Toc194674783)

[Figure S3. Baseline levels of secreted cytokines in PBMC culture supernatants before and after OIT after 48h in medium-only culture. 18](#_Toc194674784)

[Figure S4. Peanut-induced cytokine levels in PBMC culture supernatants before and after OIT after 48 h with peanut antigen. 19](#_Toc194674785)

[Figure S5. PHA-induced cytokine levels in PBMC culture supernatants before and after OIT after 48 h with PHA 20](#_Toc194674786)

[Figure S6. PHA-induced cytokine levels in PBMC culture supernatants before and after OIT, adjusted for baseline levels 21](#_Toc194674787)

[Figure S7. Estimated PBMC composition by condition and desensitization group 22](#_Toc194674788)

[Figure S8. STRING interaction network of 37 DEGs in peanut-stimulated PBMCs significantly enriched for the term “external exosome” 24](#_Toc194674789)

[Figure S9. Comparative gene set enrichment analysis for DEGs and genes linked to variation in DNA methylation from peanut-stimulated and unstimulated PBMCs 25](#_Toc194674790)

[Figure S10. Expression levels of DEGs before OIT 27](#_Toc194674791)

[Figure S11. Module-trait relationship heatmaps illustrating correlations between WGCNA co-expression modules and sampling/OIT time points 28](#_Toc194674792)

[Figure S12. Functional enrichment analysis of the “Firebrick” gene module associated with changes before versus after OIT in unstimulated PBMCs from complete responders 29](#_Toc194674793)

[Figure S13. Functional enrichment analysis of the “Darkgrey” gene module associated with changes before versus after OIT in unstimulated PBMCs from complete responders 30](#_Toc194674794)

[Figure S14. Functional enrichment analysis of the “Gainsboro” gene module associated with changes before versus after OIT in peanut-stimulated PBMCs from incomplete responders 31](#_Toc194674795)

[Figure S15. Functional enrichment analysis of the “Lightcoral” gene module associated with changes before versus after OIT in unstimulated PBMCs from incomplete responders 32](#_Toc194674796)

# Experimental methods

## Isolation of peripheral blood mononuclear cells (PBMCs)

Peripheral blood mononuclear cells (PBMCs) were isolated from heparinized venous blood by density gradient separation. The cell count was determined using Sysmex XE-2100 Hematology Analyzer (Sysmex Corporation, Kobe, Japan). The cell suspension was then adjusted to a concentration of 10x10^6^ cells/mL in RPMI medium supplemented with 10% autologous plasma).^1^

## Stimulation of PBMCs

PBMCs (2x10^6^ cells/500 μL) were subjected to three different treatments. For antigen-specific stimulation, cells were exposed to LPS-free crude peanut extract at a concentration of 50 μg/mL. For the negative control (unstimulated condition), cells were maintained in RPMI medium with 10% autologous plasma. For unspecific stimulation (positive control), cells were treated with phytohemagglutinin (PHA, 20 μg/mL). All conditions were incubated for 48h at 37°C with 5% CO_2_. After 48h of incubation, 400 µl of the cell culture supernatant was collected from each sample for further use.^1^ The PBMCs in the remaining cell culture were lysed in 200 µl of RLT buffer (Qiagen, 79216) and stored at -80°C.

## Peanut extract preparation for stimulation of PBMCs

For PBMC stimulation, peanut extract was prepared from roasted peanuts (Jumbo Peanuts, Seeberger, Hamburg, Germany) using the protocol by Burks et al.^2^ Briefly, 1200 g of peanuts were peeled, skinned and ground into a homogeneous paste. To degrease, 100 g of peanut paste were mixed with 1000 mL of pre-cooled acetone (Roth, Karlsruhe, Germany), stirred for 2.5 hours, and filtered through paper filter (Macherey-Nagel, Düren, Germany). The residue was rinsed again with 1000 mL of acetone. The filter paper containing the residue was left to dry at 4°C overnight. This process yielded 70 g of peanut powder from the initial 100 g of paste.

Small amounts of peanut powder were gradually mixed with 1x PBS (1:2, w/v) until a liquid extract was obtained. The extract was supplemented with protease inhibitor (Protease-Inhibitor-Cocktail Tablets cOmplete^TM^ EDTA-free, Roche Diagnostics, Basel, Switzerland) dissolved in 1x PBS, as recommended by the manufacturer, and incubated at 4°C overnight. Following centrifugation for 20 minutes at 3500 rpm (Sorvall RT 6000D, Thermo Scientific, Langenselbold, Germany), the upper lipid-containing phase was discarded, and the middle protein-rich phase was collected.

The protein concentration of the extract was measured using the Pierce^TM^ BCA Protein Assay Kit (Thermo Scientific, Illinois, USA), adjusted to a concentration of 2 mg/mL with PBS, aliquoted, and stored at -80°C. The concentration of lipopolysaccharides (LPS) in the extract was determined using a Limulus Amebocyte Lysate assay according to the manufacturer’s instructions. The final LPS concentration was at a cell culture-acceptable level of 0.114 EU/ml.

## Cytokine levels in cell culture supernatants

After 48 hours of incubation of PBMCs with either peanut antigen or medium alone, the concentration of IFN-γ, TNF-α, IL-2, IL-4, IL-5 and IL-10 in the cell culture supernatants (see previous section “Stimulation of PBMCs”) were measured using the BD Cytometric Bead Array Human Th1/Th2 Cytokine Kit (BD Bioscience, California, USA), as previously described.^3^ Final cytokine levels were calculated by subtracting the values from medium-only cultures from those of peanut-stimulated cultures.^1^ Cytokine levels from medium-only, peanut-stimulated, PHA-stimulated, and PHA-stimulated minus medium-only, were also analyzed.

## DNA and RNA isolation

PBMC lysates were thawed, and β-mercaptoethanol (Sigma) was added at a ratio of 1:100 to the original lysate volume. DNA isolation was performed using the Allprep DNA/RNA/miRNA Universal Kit (Qiagen, 80224). RNA was isolated using a TRIzol (Invitrogen, 10296010)/chloroform separation method. RNA extracted from the aqueous phase was further purified using the RNA Clean&Concentrator-5 kit (Zymo, R1015) and eluted in RNase-free water. Both DNA and RNA were stored at -80°C until further use. DNA quality and quantity were assessed using a NanoDrop, and RNA quality was evaluated using the Agilent 2100 Bioanalyser with a Nano chip.

## Bulk RNA sequencing and methylation array

Total RNA libraries were prepared using the TruSeq Stranded Total RNA workflow according to standard protocols (Illumina). Libraries were sequenced using the HiSeq 4000 platform to a depth of 50 million paired-end reads. Sequencing reads were aligned to the human reference genome GRCh38 using STAR version 2.6.1a. A raw count matrix was then generated, indicating the number of sequencing fragments mapped to each gene for downstream analysis.

In parallel, genomic DNA extracted from PBMC samples was treated with sodium bisulfite and subjected to genome-wide DNA methylation profiling using the Inﬁnium MethylationEPIC BeadChip.

# Statistical analysis

## Deconvolution of PBMC composition

PBMCs are composed of diverse cell types, which can influence measured DNA methylation and gene expression levels. To account for cellular heterogeneity, we implemented the deep-learning-based deconvolution tool Scaden v1.1.2.^4^ We trained Scaden on simulated bulk RNA-sequencing data derived from single-cell RNA-sequencing (scRNA-seq) data of 14 independent PBMC samples, with the aim of predicting cell composition in the bulk RNA-seq data from our study participants. The training set included 9 in-house scRNA-seq datasets from children with food allergies (7 of whom had peanut allergy; see “Generation of in-house PBMC scRNA-seq datasets” below for further details), consisting of 3 samples from unstimulated PBMCs (0 h), 3 samples after 48 h of peanut-antigen stimulation, and 3 samples after 48 h of culture in medium alone. Additionally, 5 publicly available human PBMC scRNA-seq datasets from healthy donors were included, downloaded from 10X Genomics (https://support.10xgenomics.com/­single-cell-gene-expression/­datasets): “6k”, “8k”, “20k”, “33k” and “68k”.

The single-cell datasets were pre-processed using the Python package Scanpy.^4^ All 14 PBMC scRNA-seq datasets were annotated by Azimuth^5^ (https://azimuth.hubmapconsortium.org/) at both the first (8 cell types) and second (28 cell types) resolution, using a reference PBMC dataset.^6^ For each scRNA-seq dataset, 8000 simulated bulk samples were generated by randomly selecting 10% of cells. The 14 resulting simulated bulk datasets were filtered (excluding genes with variance < 0.1), merged into a single training set (112,000 samples), and used to train a deep neural network using Scaden.

The trained model was then applied to our bulk PBMC RNA-sequencing datasets to estimate cell type composition. Estimates at the first resolution (8 cell types: CD4+, CD8+, other T cells, B-cells, natural killer cells, monocytes, dendritic cells, and others) were included as covariates to adjust downstream statistical models. Estimates at the second resolution (28 cell types) are shown in Figure S7.

## Generation of in-house PBMC scRNA-seq datasets

### PBMCs isolation and stimulation

Peripheral blood samples were collected from six food-allergic individuals, including four with peanut allergy, enrolled in the *CHildhood Allergy and Tolerance: bioMarkers and Predictors (CHAMP)* study.^7^ PBMCs were isolated via density gradient centrifugation using Biocoll Separating Solution (L6113 - Merck) layered in SepMate-15™ tubes (StemCell Technologies). PBMCs of 3 peanut allergic individuals were either stimulated with crude peanut extract or left unstimulated in RPMI 1640 Medium (Gibco) for 48 hours at a density of 2 x 10^6^ cells/mL.

### PBMC processing on the 10X Genomics platform and sequencing

Nine single-cell suspensions, derived from six patients, were loaded on the Chromium Controller (10X Genomics) with targeted cell recovery of 10000 cells per sample. Gene expression libraries were prepared according to the manufacturer’s protocol using the Chromium NextGEM 3’ Single Cell Reagent Kits (10X Genomics). Library and cDNA quality were assessed using the Bioanalyzer High-Sensitivity DNA Analysis Kits (Agilent) and KAPA Library Quantification Kits (Roche). The completed libraries were sequenced on Illumina NovaSeq 6000 machines, targeting 50,000 reads per cell.

### Data processing and downstream analysis

Raw BCL files generated by the NovaSeq 6000 (Illumina) were converted into FASTQ files using bcl2fastq. Samples were mapped to the human reference genome GRCh38 using the CellRanger suite v6.1.2 (10X Genomics). CellRanger summary statistics were used to assess mapping quality and other quality control metrics. The resulting filtered feature-barcode matrices were analysed using Azimuth^5^, as described in the previous section “Deconvolution of PBMC composition”.

## Gene expression analysis

Differentially expressed genes (DEGs) were identified from RNA-sequencing count data using DESeq2.^8^ Ensembl gene IDs were converted to HGNC symbols, and genes located on sex chromosomes were excluded. Counts-per-million (CPM) values were calculated using edgeR^9^ to filter out lowly expressed genes. Genes were retained for analysis if their expression was above 0.5 CPM in at least 5% of all samples. After quality control, expression levels of 15,046 genes were tested for their association with oral immunotherapy (OIT) outcomes:

i) ‘*complete versus incomplete desensitization*’ (binary), and

ii) ‘*increment in tolerated peanut protein after OIT*’ (quantitative).

Analyses were performed in peanut-stimulated, phytohemagglutinin (PHA)-stimulated, and unstimulated PBMCs.

### Primary analysis: Overall effects on desensitisation

Our **primary analysis** aimed to identify DEGs associated with OIT outcomes by testing the combined effect of OIT outcome group and its interaction with time. A likelihood ratio test (LRT) was applied by comparing the following two models, where the difference (*i.e.* the tested terms by DESeq2) is highlighted in bold:

- Full model: ~ Covariates + OIT_timepoint + **OIT_outcome + OIT_outcome:OIT_timepoint**
- Reduced model: ~ Covariates + OIT_timepoint

In this context, “OIT_outcome” refers to either the binary OIT outcome (complete vs. incomplete desensitisation) or the quantitative change in tolerated peanut protein, and “OIT_timepoint” refers to the time of sample collection (before vs. after OIT). All models were adjusted for covariates: age, sex, RNA integrity number (RIN), PBMC composition, and one factor of unwanted variation to account for unmodeled random effects not directly included in the model (e.g. batch effects or other technical sources of variation).

Effect sizes were reported as the combined log_2_ fold change from the two terms tested by the LRT. Our primary analysis identifies genes whose expression is associated either with the treatment outcome itself (main effect) or with a change over time that differs by outcome group (interaction effect), or both.

### Subgroup analyses and specific models

To complement our primary analysis, we performed a series of subgroup analyses targeting specific biological questions (see Figure 1D). Only genes with sufficiently high counts, retained after applying the filterByExpr() function from edgeR, were included in each comparison.

1. Pre-OIT samples: Predictive biomarkers in verum participants.

Aim: To identify gene expression differences present before treatment that may predict OIT success and could serve as predictive biomarkers.

Model tests: The **main effect** of OIT outcome (binary or quantitative) before therapy.

- - Model: ~ Covariates + OIT_outcome

1. **Post-OIT samples: Response differences in verum participants**

*Aim*: To assess how OIT-induced gene expression differs between outcome groups.

*Model tests*: The main effect of OIT outcome (binary or quantitative) after therapy.

- Model: ~ Covariates + OIT_outcome

1. **Complete responders: Before vs. after OIT**

*Aim*: To capture gene expression changes over time in participants who achieved complete desensitization.

*Model tests*: The main effect of OIT timepoint (pre vs. post) within complete responders.

- Model: ~ Covariates + OIT_timepoint

1. **Incomplete responders: Before vs. after OIT**

*Aim*: To capture gene expression changes over time in participants with incomplete desensitization (who did not achieve a complete desensitization to 4.5g of peanut protein).

*Model tests*: The main effect of OIT timepoint (pre vs. post) within incomplete responders.

- Model: ~ Covariates + OIT_timepoint

1. **Pre-OIT samples: Verum vs. placebo groups**

*Aim*: To assess baseline gene expression differences between treatment arms.

*Model tests*: The main effect of treatment group (verum vs. placebo) at baseline.

- Model: ~ Covariates + OIT_Study_Arm

1. **Post-OIT samples: Verum vs. placebo groups**

*Aim*: To isolate gene expression changes specifically attributable to OIT, independent of placebo effects.

*Model tests*: The main effect of treatment group after OIT.

- Model: ~ Covariates + OIT_Study_Arm

All subgroup analyses were performed separately for peanut-stimulated, PHA-stimulated, and unstimulated PBMCs. Models were adjusted for the same covariates as in the primary analysis. A Wald test (rather than an LRT) was used to assess the relevant term in each model. *P*-values were adjusted using the Benjamini–Hochberg false discovery rate (FDR) procedure with a default alpha of 0.1 in DESeq2. Factors of unwanted variation from RNA-seq data were estimated from each specific subgroup using RUVSeq package^10^.

## Weighted Gene Co-expression Network Analysis (WGCNA)

To further explore relationships between gene co-expression patterns and OIT outcomes, Weighted Gene Co-expression Network Analysis (WGCNA) was performed on RNA-seq data from PMBC samples from the 38 participants in the active arm. This analysis aimed to identify modules of co-expressed genes associated with OIT response. Samples were stratified into four subsets:

- **Before Treatment**: To capture baseline gene expression profiles prior to OIT.
- **After Treatment**: To assess gene expression changes following OIT.
- **Complete Responders**: To assess gene expression changes from before to after OIT associated with a positive OIT response.
- **Incomplete Responders**: To assess gene expression changes from before to after OIT linked with a lack of positive response to OIT.

Analyses were conducted separately for peanut-stimulated and unstimulated PBMCs. Normalization and variance stabilization were performed using DESeq2, including library size normalization and variance-stabilizing transformation (VST) applied separately to each subset.

WGCNA was applied to each subset to identify gene modules and their correlations with treatment response (complete vs. incomplete responders) or OIT timepoint (before vs. after OIT). A weighted co-expression network was constructed based on pairwise gene correlations. Modules were identified via hierarchical clustering and dynamic tree cutting (resolution parameter set at 0.25) to control module granularity.

Correlations between modules and traits were tested, with significance determined by Bonferroni correction based on the number of detected modules. Module preservation analysis was used to assess robustness across subsets. These analyses provided insight into how co-expressed gene networks relate to immune tolerance, OIT-induced changes, and treatment response variability.

## Epigenome-wide association analysis of CpGs and differentially methylated regions (DMRs)

Meffil was used to pre-process, normalize, and analyze DNA methylation data.^11^ Briefly, raw intensity values underwent pre-processing and quantile-normalization, with adjustment for 15 principal components to control for technical variation. Functional normalization accounted for confounding effects using slide, sentrix row and sample ID as random effects, and timepoint, condition, sex, standardized age and estimated cell composition as fixed effects.

Quality control thresholds were applied: CpGs with a detection *p-*value > 0.01 in more than 5% of samples, or with a bead count < 3 were excluded. Post-OIT samples from one participant were not available. CpGs located on sex chromosomes or known to show non-specific binding were also removed. A total of 791,084 CpGs passed QC and were retained for further analysis. Independent surrogate variables were estimated and included to adjust for unmodeled variation.

DNA methylation analyses were performed for two conditions (medium and peanut-stimulated) and two phenotypes: the binary OIT outcome “*complete versus incomplete desensitization*” and the quantitative trait “*increase in tolerated peanut protein after OIT*”. Differentially methylated probes (DMPs) were identidied using a genome-wide significance threshold of 9x10^-8^, which adequately controls the false positive rate for llumina EPIC arrays.^12^ Tto empirically assess robustness of our findings, we conducted 1,000 genome-wide DNA methylation analyses on randomly permuted phenotypes, confirming the reliability of observed findings (Figure S2).

DMPs were annotated to genes using an annotation package designed for llumina EPIC methylation arrays^13^ which maps probes to UCSC reference genes between 1500 bp upstream of the transcription start site and the polyadenylation signal.

Because DNA methylation typically occurs across multiple adjacent CpGs within regulatory regions, we used the Comb-p algorithm to identify differentially methylated regions (DMRs) by combining spatially correlated *p-*values.^13^ DMR were defined using a seed *p-*value of 0.05, a maximum distance of 750 bp between CpGs, and a minimum of 3 CpGs per DMR. Statistical significance of DMRs was determined using Sidak correction for multiple testing, with a significance threshold set at *p-*value < 0.05. DMR coordinates were converted to hg38, and gene annotations were obtained using BiomaRt, including gene products overlapping or located within 5 kb of significant DMRs.

### Subgroup analyses and specific models

The subgroup analyses described previously for gene expression were similarly applied to DNA methylation data, using the same pre-processing, normalization, and modeling pipeline in Meffil and Comb-p as outlined above, in order to identify DMPs and DMRs across the specified comparisons.

### Integration of gene expression with DNA methylation changes

We used our own DNA methylation and gene expression data to identify expression quantitative trait methylation sites (cis-eQTMs), defined as CpGs whose methylation level correlates with the expression level of a gene. To do this, all DMPs were paired with DEGs located within a ±1.5 Mb range. We then correlated levels of CpG methylation and gene expression among samples from the unstimulated and peanut-stimulated conditions at each OIT time point, resulting in four datasets. We used Kendall’s correlation, and a *p <* 0.05 after false discovery rate adjustment, to define eQTMs.

To investigate how DNA methylation may regulate gene expression and OIT outcomes, we looked for genes with supporting evidence from both methylation and transcriptomic data. This was done by overlapping all DEGs with genes annotated to DMPs, DMRs and eQTMs. A hypergeometric test was used to determine whether the overlap between DEGs and genes with differentially methylated patterns was significantly larger than expected by chance.

### Functional enrichment analysis

Based on the source of association with the OIT response, we defined two sets of genes from our primary analysis among unstimulated and peanut-stimulated PBMCs: 184 DEGs and 1001 genes linked to significant patterns of methylation. We performed functional enrichment analysis on both gene sets using the Enrichr platform,^14,15^ with access to numerous databases to inspect enrichment in cell types (libraries: Immune cells of Descartes Cell Types and Tissue 2021 and HuBMAP ASCTplusB augmented 2022), diseases (library: GWAS Catalog 2023; only disease diagnoses considered), ontologies (libraries: Gene Ontology (2023): biological process, cellular component, molecular function, and Jensen COMPARTMENTS), pathways (libraries: BioPlanet 2019, KEGG 2021, WikiPathways 2024, and Reactome 2024), and transcription factors (library: ChEA 2022 (primary human tissues/cells). Only terms that included at least 10 genes and had more than 3 overlapping genes were considered. An FDR-adjusted *p-*value < 0.05 was established as significant. To assess the robustness of the findings, empirical P-values were estimated by conducting 1000 permutations of the enrichment analysis using random set of gene for either unstimulated or peanut-stimulated PBMCs. Additionally, we applied a hypergeometric test to evaluate the probability that the immune cells from HuBMAP ASCTplusB identified by DEGs were also detected by genes linked to differentially methylated patterns. Functional enrichment analysis was similarly applied to gene sets from each specific condition (either unstimulated or peanut-stimulated PBMCs), as well as to co-expressed genes from significant gene modules identified by WGCNA.

# Supplementary References

1. Blumchen K, Ulbricht H, Staden U, et al. Oral peanut immunotherapy in children with peanut anaphylaxis. *J Allergy Clin Immunol*. 2010;126(1):83-91 e81.

2. Burks AW, Williams LW, Connaughton C, Cockrell G, O'Brien TJ, Helm RM. Identification and characterization of a second major peanut allergen, Ara h II, with use of the sera of patients with atopic dermatitis and positive peanut challenge. *J Allergy Clin Immunol*. 1992;90(6 Pt 1):962-969.

3. Blumchen K, Beder A, Beschorner J, et al. Modified oral food challenge used with sensitization biomarkers provides more real-life clinical thresholds for peanut allergy. *J Allergy Clin Immunol*. 2014;134(2):390-398.

4. Menden K, Marouf M, Oller S, et al. Deep learning-based cell composition analysis from tissue expression profiles. *Sci Adv*. 2020;6(30):eaba2619.

5. Hao Y, Hao S, Andersen-Nissen E, et al. Integrated analysis of multimodal single-cell data. *Cell*. 2021;184(13):3573-3587.e3529.

6. Stuart T, Butler A, Hoffman P, et al. Comprehensive Integration of Single-Cell Data. *Cell*. 2019;177(7):1888-1902.e1821.

7. Eckert JK, Kahle J, Bock A, et al. CHildhood Allergy and tolerance: Biomarkers and Predictors (CHAMP) and quality of life. *Pediatr Allergy Immunol*. 2022;33(1):e13652.

8. Love MI, Huber W, Anders S. Moderated estimation of fold change and dispersion for RNA-seq data with DESeq2. *Genome Biol*. 2014;15(12):550.

9. Robinson MD, McCarthy DJ, Smyth GK. edgeR: a Bioconductor package for differential expression analysis of digital gene expression data. *Bioinformatics*. 2010;26(1):139-140.

10. Risso D, Ngai J, Speed TP, Dudoit S. Normalization of RNA-seq data using factor analysis of control genes or samples. *Nat Biotechnol*. 2014;32(9):896-902.

11. Min JL, Hemani G, Davey Smith G, Relton C, Suderman M. Meffil: efficient normalization and analysis of very large DNA methylation datasets. *Bioinformatics*. 2018;34(23):3983-3989.

12. Mansell G, Gorrie-Stone TJ, Bao Y, et al. Guidance for DNA methylation studies: statistical insights from the Illumina EPIC array. *BMC Genomics*. 2019;20(1):366.

13. Hansen KD. IlluminaHumanMethylationEPICanno.ilm10b2.hg19: Annotation for Illumina's EPIC methylation arrays. 2016;

14. Pedersen BS, Schwartz DA, Yang IV, Kechris KJ. Comb-p: software for combining, analyzing, grouping and correcting spatially correlated P-values. *Bioinformatics*. 2012;28(22):2986-2988.

15. Kuleshov MV, Jones MR, Rouillard AD, et al. Enrichr: a comprehensive gene set enrichment analysis web server 2016 update. *Nucleic Acids Res*. 2016;44(W1):W90-97.

16. Xie Z, Bailey A, Kuleshov MV, et al. Gene Set Knowledge Discovery with Enrichr. *Curr Protoc*. 2021;1(3):e90.

# Supplementary Figures


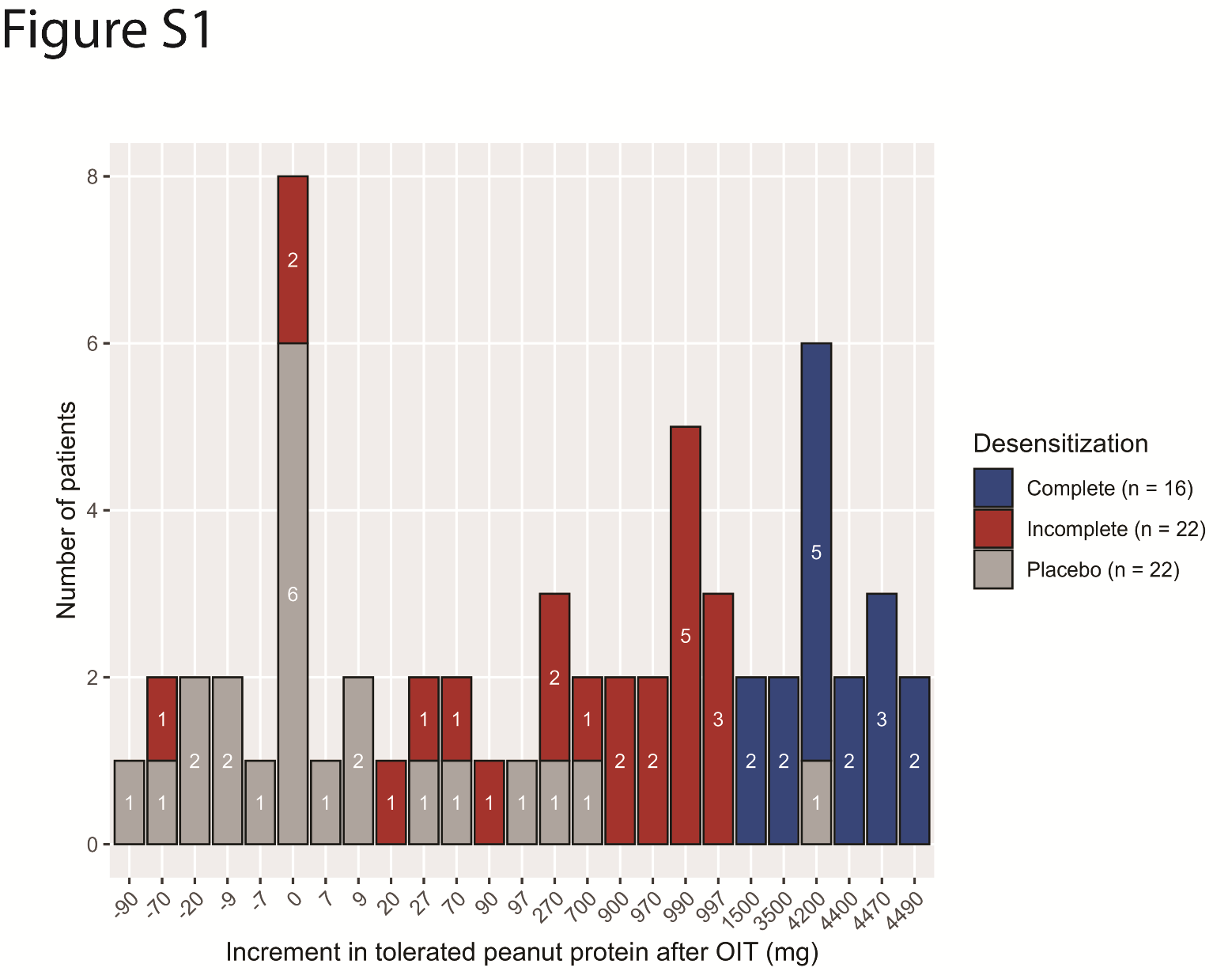


Figure S1. Increments in tolerated peanut protein after OIT. Complete responders (n = 16), incomplete responders (n = 22), and placebo participants (n = 22) are shown in blue, red, and grey, respectively. The x-axis represents the increase tolerated peanut protein between the initial oral food challenge before OIT and the final oral food challenge after OIT. For verum participants, the median increment was 993.5 mg, with an interquartile range of 3450 mg. The y-axis indicates the number of patients who achieved the corresponding increments. This variable was used as the quantitative trait in gene expression and DNA methylation analyses. Abbreviations: OIT, oral immunotherapy.


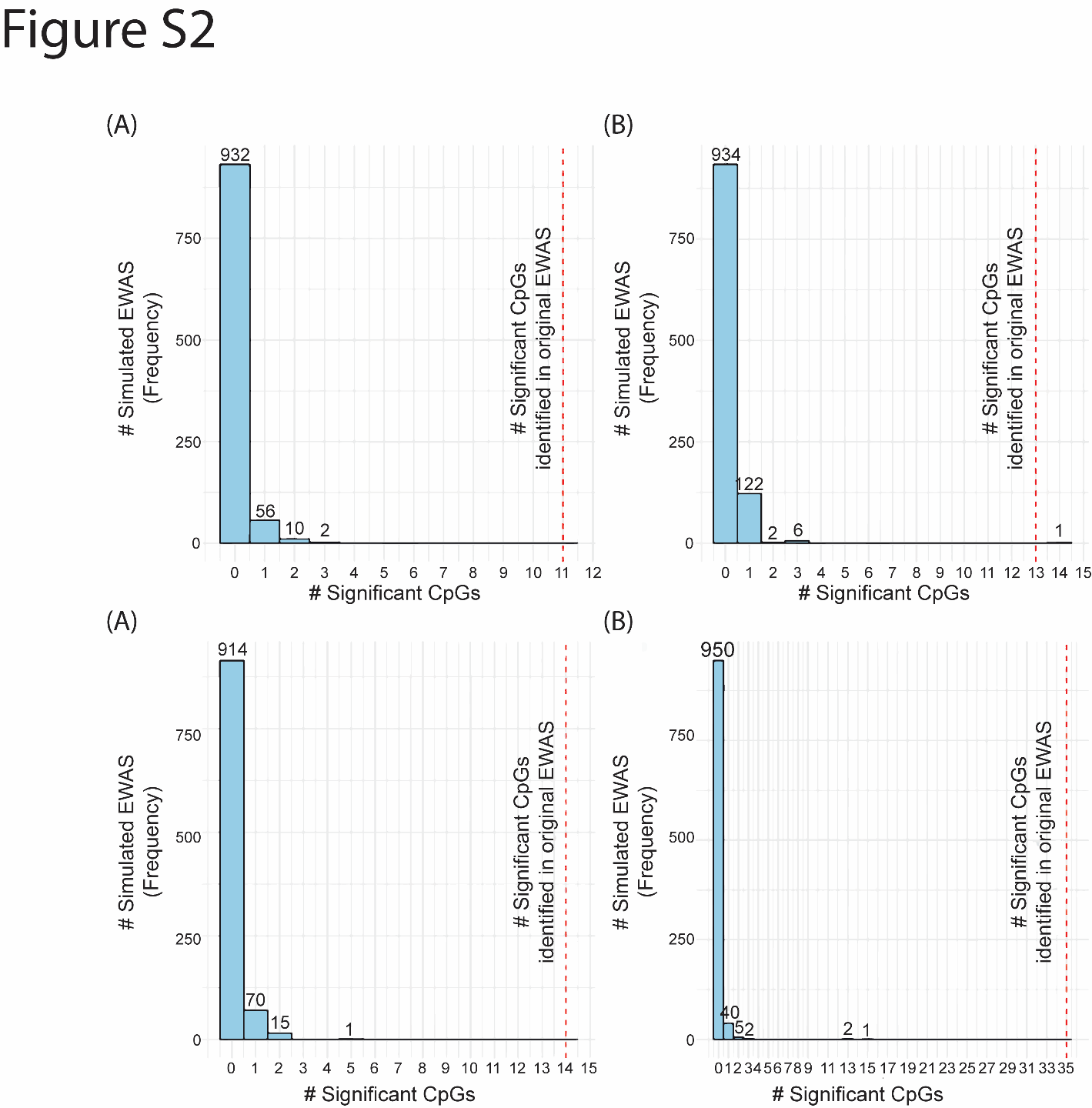


Figure S2. Histogram of simulated epigenome-wide analysis. Histograms show the number of CpGs detected at p-value < 9x10^-8^ (the genome-wide significant threshold used to control the false positive rate in the original epigenome-wide analysis) after permuting the results of the individual DNA methylation probe analysis 1000 times in unstimulated PBMCs for (A) the binary phenotype “*complete versus incomplete desensitization*” and (B) the quantitative phenotype “*increment in tolerated peanut protein after OIT*”, as well as in peanut-stimulated PBMCs (C) for the binary and (D) quantitative phenotype. The x-axis represents the number of significant DMPs detected in the simulations, and the y-axis indicates the frequency of permutations yielding the corresponding number of significant findings. The dashed red line marks the number of significant DMPs detected in our primary (non-permuted) analysis. Abbreviations: EWAS, epigenome-wide association study; CpGs, cytosine-phosphate-guanine sites, PBMCs, peripheral blood mononuclear cells; OIT, oral immunotherapy; DMPs, differentially methylated probes.


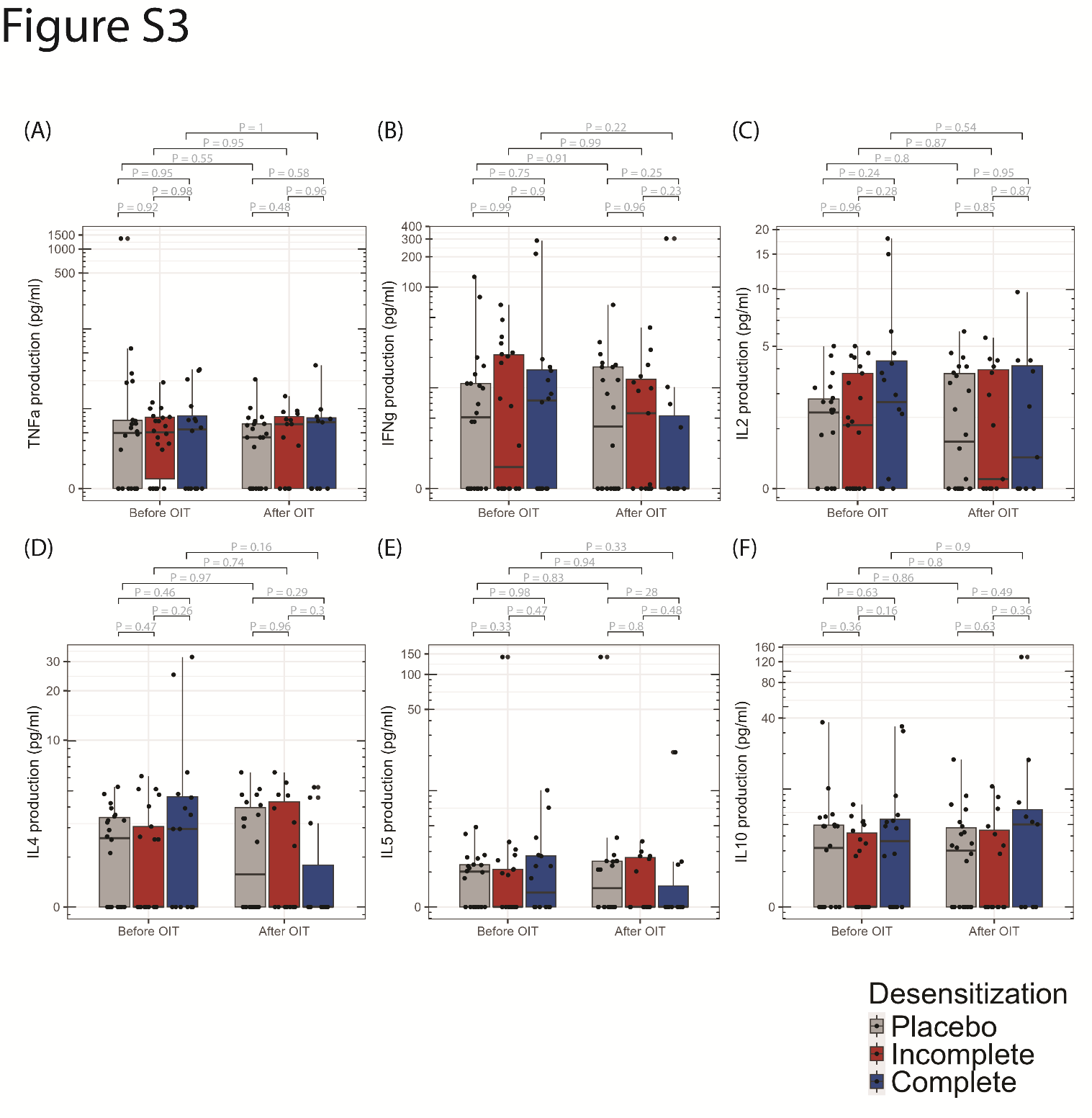
Figure S3. Baseline levels of secreted cytokines in PBMC culture supernatants before and after OIT after 48h in medium-only culture. Levels of cytokines: (A) TNF-α, (B) IFN-γ, (C) IL-2, (D) IL-4, (E) IL-5, and (F) IL-10, were measured in unstimulated PBMC culture supernatants collected after 48 hours of medium-only cultures. Data are shown for 49 participants, comprising 16 complete responders (blue), 22 incomplete responders (red), and 22 placebos participants (grey). For 11 participants who crossed over to the active arm after receiving placebo, cytokine levels after verum treatment were unavailable; thus, comparisons after OIT included 11 complete and 16 incomplete responders. All cytokine values are displayed on a logarithmic scale. Statistical significance was assessed using Wilcoxon signed-rank tests with continuity correction for pairwise comparisons within desensitization groups (before versus after OIT) and Kruskal-Wallis one-way analysis of variance on ranks for comparisons between groups. Non-significant adjusted *p*-values after correction for multiple testing (*p* < 0.0083) are indicated in grey. Box plots depict median (central line), interquartile range (box), and 5th to 95th percentiles (whiskers). Abbreviations: OIT, oral immunotherapy; PBMCs, peripheral blood mononuclear cells; IL, interleukin; TNF, tumor necrosis factor; IFN, interferon


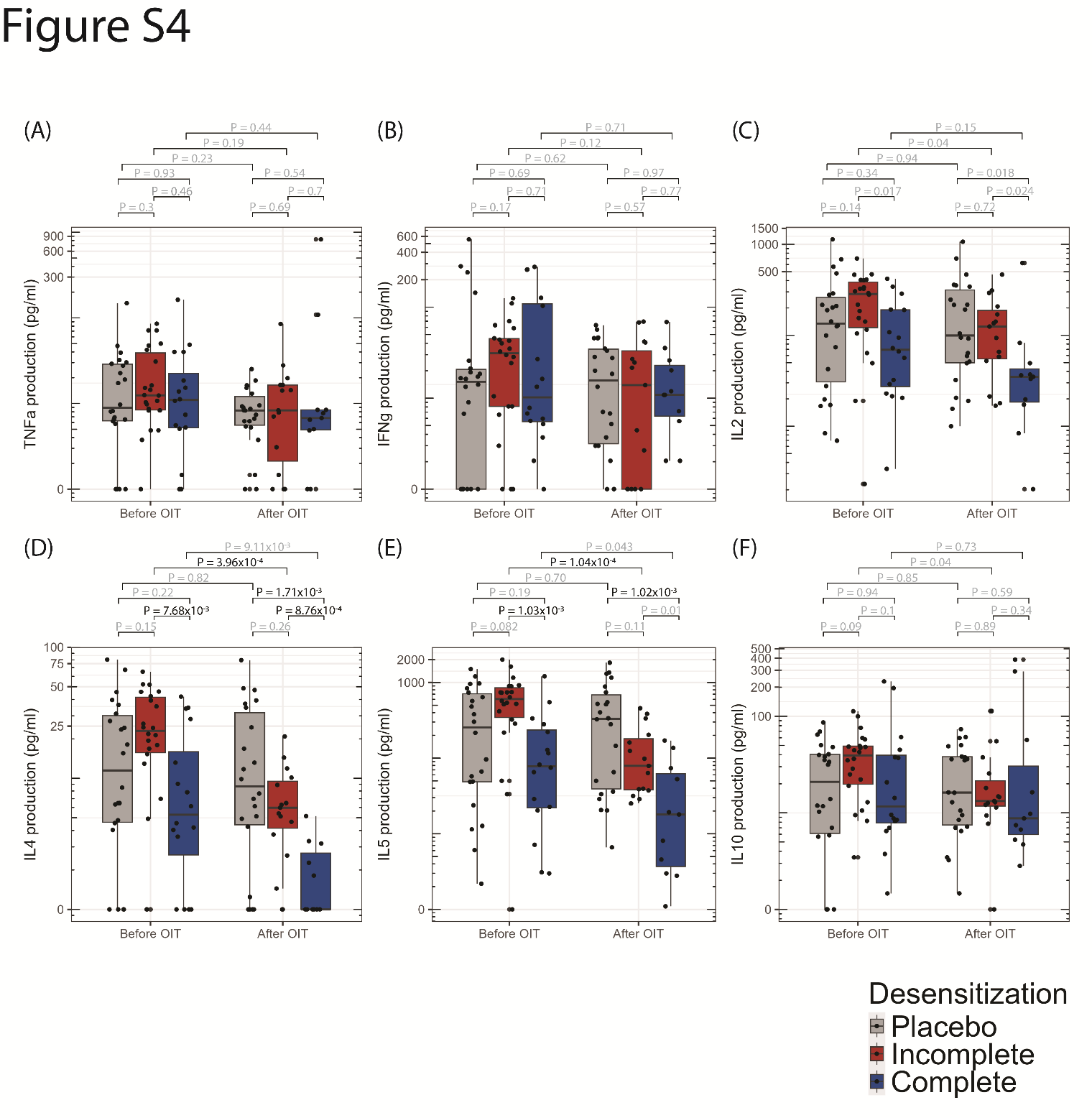
Figure S4. Peanut-induced cytokine levels in PBMC culture supernatants before and after OIT after 48 h with peanut antigen. Levels of cytokines: (A) TNF-α, (B) IFN-γ, (C) IL-2, (D) IL-4, (E) IL-5, and (F) IL-10, were measured in peanut-stimulated PBMC culture supernatants collected after 48 hours of culture. No subtraction of baseline (medium-only) levels was performed. Data are shown for 49 participants, comprising 16 complete responders (blue), 22 incomplete responders (red), and 22 placebo participants (grey). For 11 participants who crossed over to the active arm after receiving placebo, cytokine levels after verum treatment were unavailable; thus, comparisons after OIT included 11 complete and 16 incomplete responders. All cytokine values are displayed on a logarithmic scale. Statistical significance was assessed using Wilcoxon signed-rank tests with continuity correction for pairwise comparisons within desensitization groups (before versus after OIT) and Kruskal–Wallis one-way analysis of variance on ranks for comparisons between groups. Non-significant adjusted *p*-values after correction for multiple testing (*p* < 0.0083) are indicated in grey. Box plots depict median (central line), interquartile range (box), and 5th to 95th percentiles (whiskers). **Abbreviations:** OIT, oral immunotherapy; PBMCs, peripheral blood mononuclear cells; IL, interleukin; TNF, tumor necrosis factor; IFN, interferon.


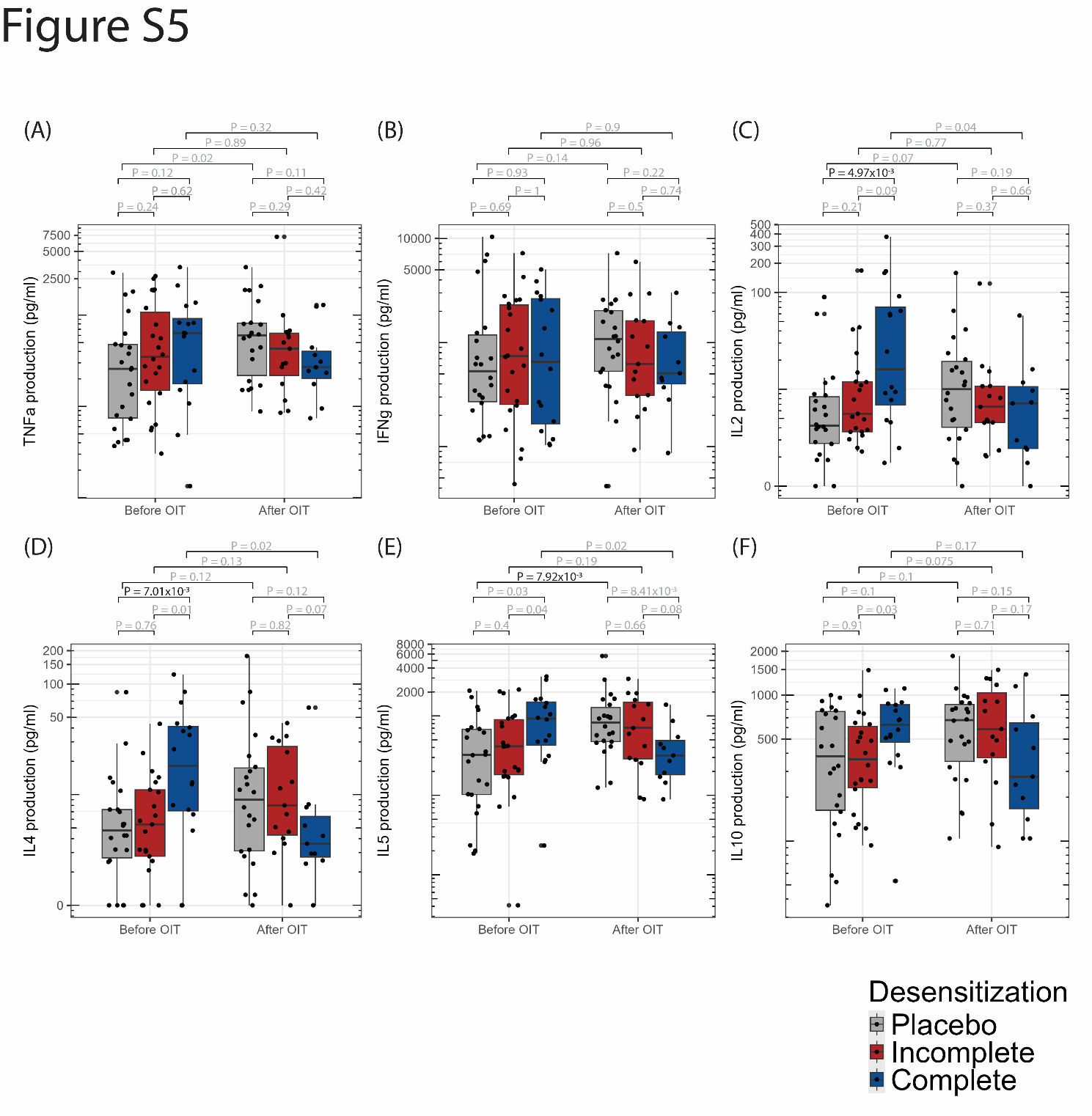
Figure S5. PHA-induced cytokine levels in PBMC culture supernatants before and after OIT after 48 h with PHA. Levels of cytokines: (A) TNF-α, (B) IFN-γ, (C) IL-2, (D) IL-4, (E) IL-5, and (F) IL-10, were measured in PHA-stimulated PBMC culture supernatants collected after 48 hours of culture. No subtraction of baseline (medium-only) levels was performed. Data are shown for 49 participants, comprising 16 complete responders (blue), 22 incomplete responders (red), and 22 placebo participants (grey). For 11 participants who crossed over to the active arm after receiving placebo, cytokine levels after verum treatment were unavailable; thus, comparisons after OIT included 11 complete and 16 incomplete responders. All cytokine values are displayed on a logarithmic scale. Statistical significance was assessed using Wilcoxon signed-rank tests with continuity correction for pairwise comparisons within desensitization groups (before versus after OIT) and Kruskal–Wallis one-way analysis of variance on ranks for comparisons between groups. Non-significant adjusted p-values after correction for multiple testing (p < 0.0083) are indicated in grey. Box plots depict median (central line), interquartile range (box), and 5th to 95th percentiles (whiskers). A**bbreviations:** OIT, oral immunotherapy; PHA, phytohemagglutinin; PBMCs, peripheral blood mononuclear cells; IL, interleukin; TNF, tumor necrosis factor; IFN, interferon.


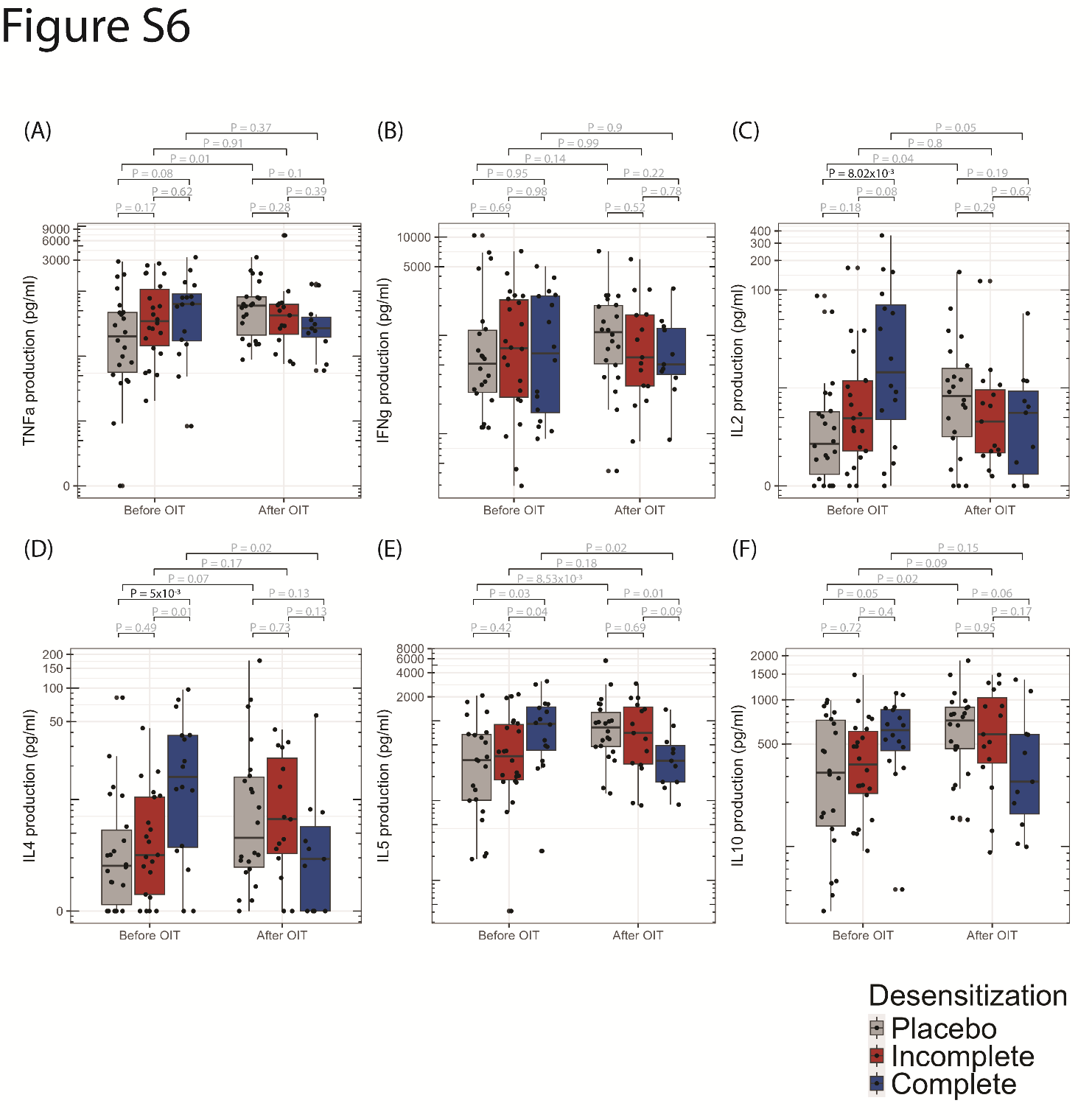
Figure S6. PHA-induced cytokine levels in PBMC culture supernatants before and after OIT, adjusted for baseline levels. Levels of cytokine responses induced by PHA stimulation—(A) TNF-α, (B) IFN-γ, (C) IL-2, (D) IL-4, (E) IL-5, and (F) IL-10—were measured in PBMC culture supernatants collected after 48 hours of stimulation and adjusted for baseline levels without stimulation (medium-only). Data are shown for 49 participants, comprising 16 complete responders (blue), 22 incomplete responders (red), and 22 placebo participants (grey). For 11 participants who crossed over to the active arm after receiving placebo, cytokine levels after verum treatment were unavailable; thus, comparisons after OIT included 11 complete and 16 incomplete responders. All cytokine values are displayed on a logarithmic scale. Statistical significance was assessed using Wilcoxon signed-rank tests with continuity correction for pairwise comparisons within desensitization groups (before versus after OIT) and Kruskal–Wallis one-way analysis of variance on ranks for comparisons between groups. Non-significant adjusted p-values after correction for multiple testing (p < 0.0083) are indicated in grey. Box plots depict median (central line), interquartile range (box), and 5th to 95th percentiles (whiskers). **Abbreviations:** OIT, oral immunotherapy; PHA, phytohemagglutinin; PBMCs, peripheral blood mononuclear cells; IL, interleukin; TNF, tumor necrosis factor; IFN, interferon.


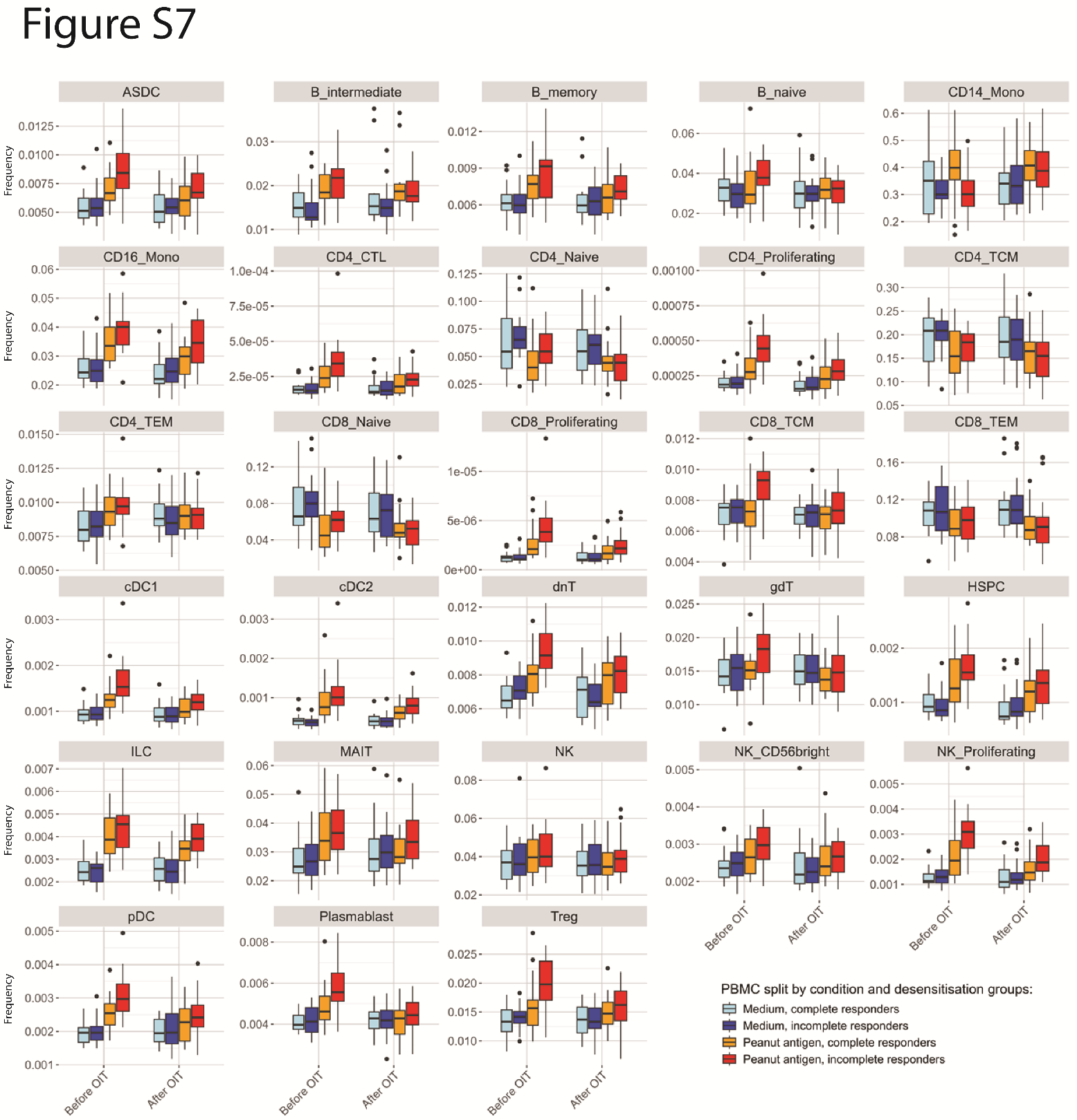


Figure S7. Estimated PBMC composition by condition and desensitization group. Boxplots display the estimated proportions of 28 distinct immune cell types in PBMC samples from study participants in the active arm, categorized by stimulation condition and desensitization response: unstimulated PBMCs from complete responders (light blue), unstimulated PBMCs from incomplete responders (dark blue), peanut-stimulated PBMCs from complete responders (orange), and peanut-stimulated PBMCs from incomplete responders (red). The y-axis shows the estimated frequency of each cell type (range: 0 to 1). No significant differences were observed between OIT desensitization groups (complete vs. incomplete responders), either in medium or under peanut stimulation, after correction for multiple testing (adjusted p-value threshold = 1.79x10^-3^). Abbreviations: ASDC, AXL+ dendritic cell; B intermediate, intermediate B cell; B memory, memory B cell; B naive, naive B cell; CD14 Mono, CD14+ monocyte; CD16 Mono, CD16+ monocyte; CD4 CTL, CD4+ cytotoxic T cell; CD4 Naive, CD4+ naive T cell; CD4 Proliferating, CD4+ proliferating T cell; CD4 TCM, CD4+ central memory T cell; CD4 TEM, CD4+ effector memory T cell; CD8 Naive, CD8+ naive T cell; CD8 Proliferating, CD8+ proliferating T cell; CD8 TCM, CD8+ central memory T cell; CD8 TEM, CD8+ effector memory T cell; cDC1, conventional dendritic cell 1; cDC2, conventional dendritic cell 2; dnT, double-negative T cell; gdT, gamma-delta T cell; HSPC, hematopoietic stem and progenitor cell; ILC, innate lymphoid cell; MAIT, mucosal associated invariant T cell; NK, natural killer cell; NK_CD56bright, CD56-bright natural killer cell; NK Proliferating, proliferating natural killer cell; pDC, plasmacytoid dendritic cell; Treg, regulatory T cell; PBMC, peripheral blood mononuclear cells; OIT, oral immunotherapy.


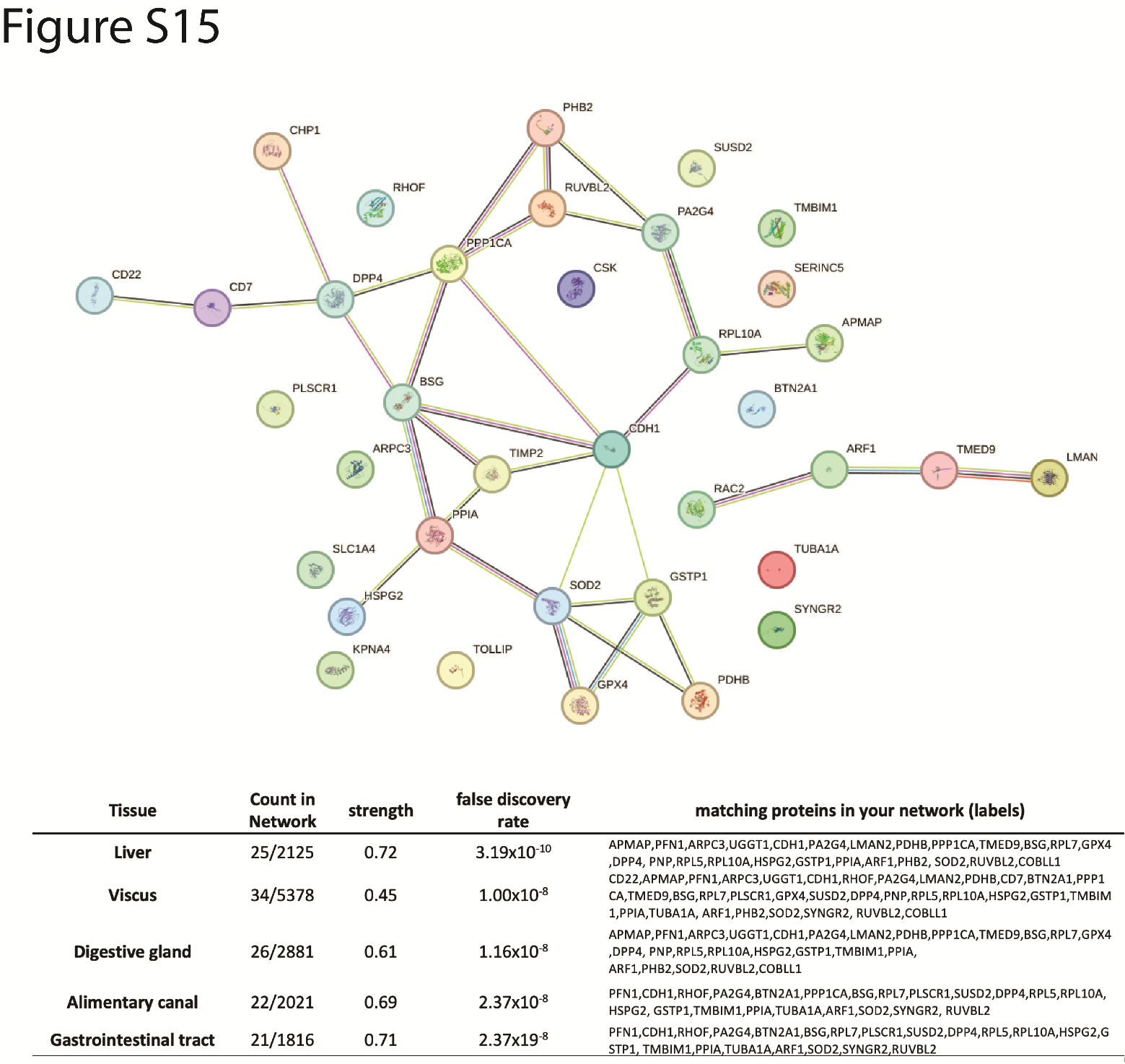


Figure S8. STRING interaction network of 37 DEGs in peanut-stimulated PBMCs significantly enriched for the term “external exosome”. Nodes represent proteins and dotted edges delineate clusters. Edge colors indicate interaction evidence: curated databases (light blue), experimental evidence (purple), gene neighborhood (green), gene fusions (red), gene co-occurrence (dark blue), text mining (yellow-green), co-expression (black), and protein homology (light violet). The top 10 functionally enriched tissue expression terms are also shown: *Count in Network* indicates the number of proteins annotated for a specific tissue term (matching proteins in the network are shown); *Strength* (log_10_[observed/expected]) reflects the magnitude of enrichment; *Signal* is the weighted harmonic mean of observed/expected and −log(FDR), balancing g term size and significance; *False Discovery Rate (FDR)* provides Benjamini–Hochberg adjusted enrichment significance.


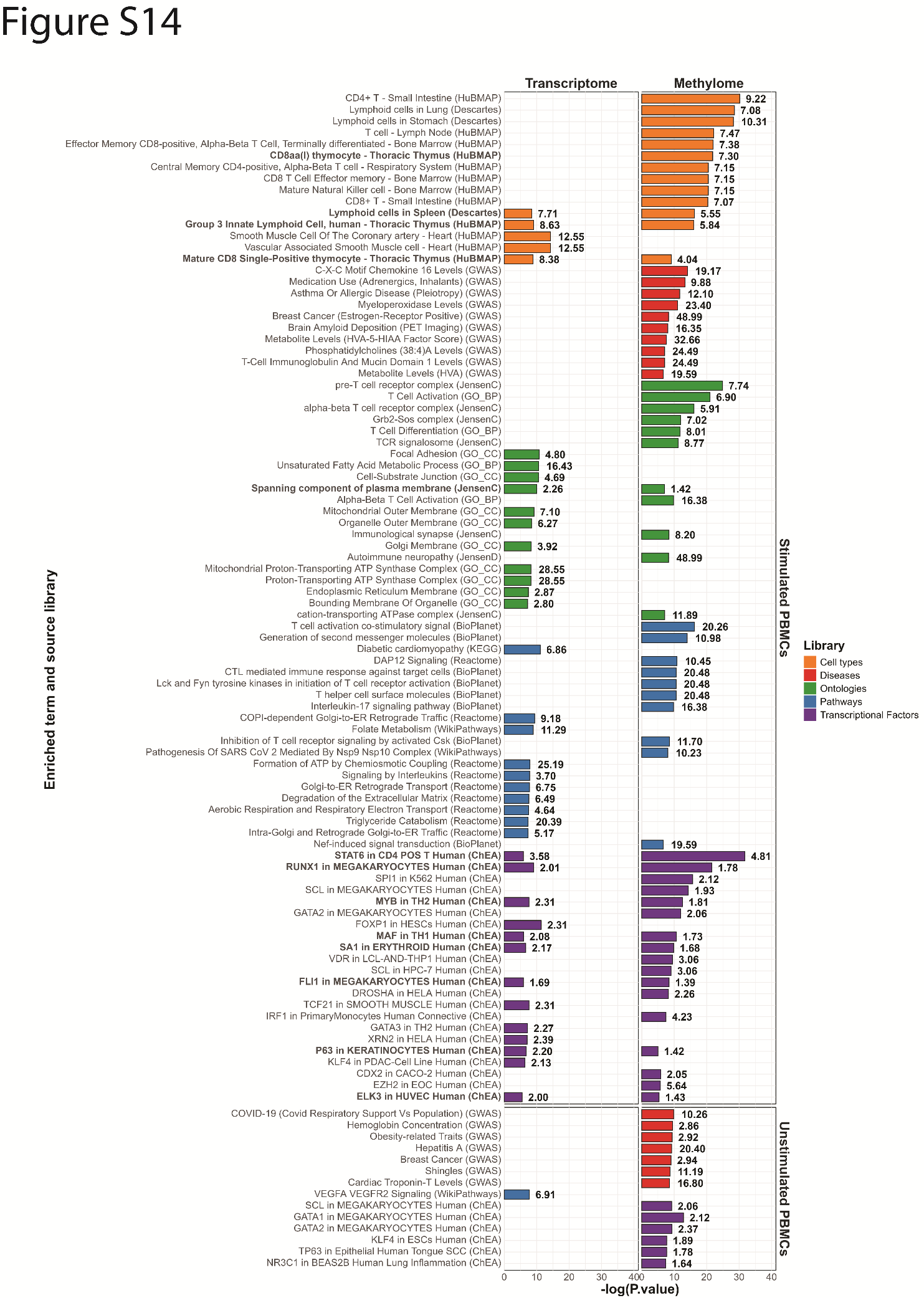
Figure S9. Comparative gene set enrichment analysis for DEGs and genes linked to variation in DNA methylation from peanut-stimulated and unstimulated PBMCs. This figure displays top 10 gene sets based on odd ratio, plus any additional terms overlapping across RNA and DNA methylation datasets, identified from four distinct datasets: 142 DEGs found in peanut-stimulated PBMCs (top-left), 46 DEGs found in unstimulated PBMCs (bottom-left), 903 genes annotated to DNA methylation differences identified in peanut-stimulated PBMCs (top-right), and 425 genes annotated to DNA methylation differences identified in unstimulated PBMCs (bottom-right). Terms are grouped into functional domains: Cell Types, Ontologies, Pathways and Transcription Factors, Diseases. The library from which each enriched term was identified is shown in parenthesis. Gene set enrichment significance is indicated by –log_10_ *p*-values. Odds ratios are shown in bold at the end of each bar. Complete enrichment results are provided in Supplementary Tables S12 and S24. Abbreviations: DEGs, differentially expressed genes; PBMCs, peripheral blood mononuclear cells; HuBMAP, Human BioMolecular Atlas Program; Descartes, Descartes Cell Types and Tissue; GWAS, Genome-Wide Association Study Catalog; JensenC, Jensen COMPARTMENTS; GO_BP, Gene Ontology Biological Process; GO_MF, Gene Ontology Molecular Function; GO_CC, Gene Ontology Cellular Component; KEGG, Kyoto Encyclopedia of Genes and Genomes; BioPlanet, NIH BioPlanet Pathway Database; WikiPathways, WikiPathways Pathway Database; ChEA, ChIP-X Enrichment Analysis.


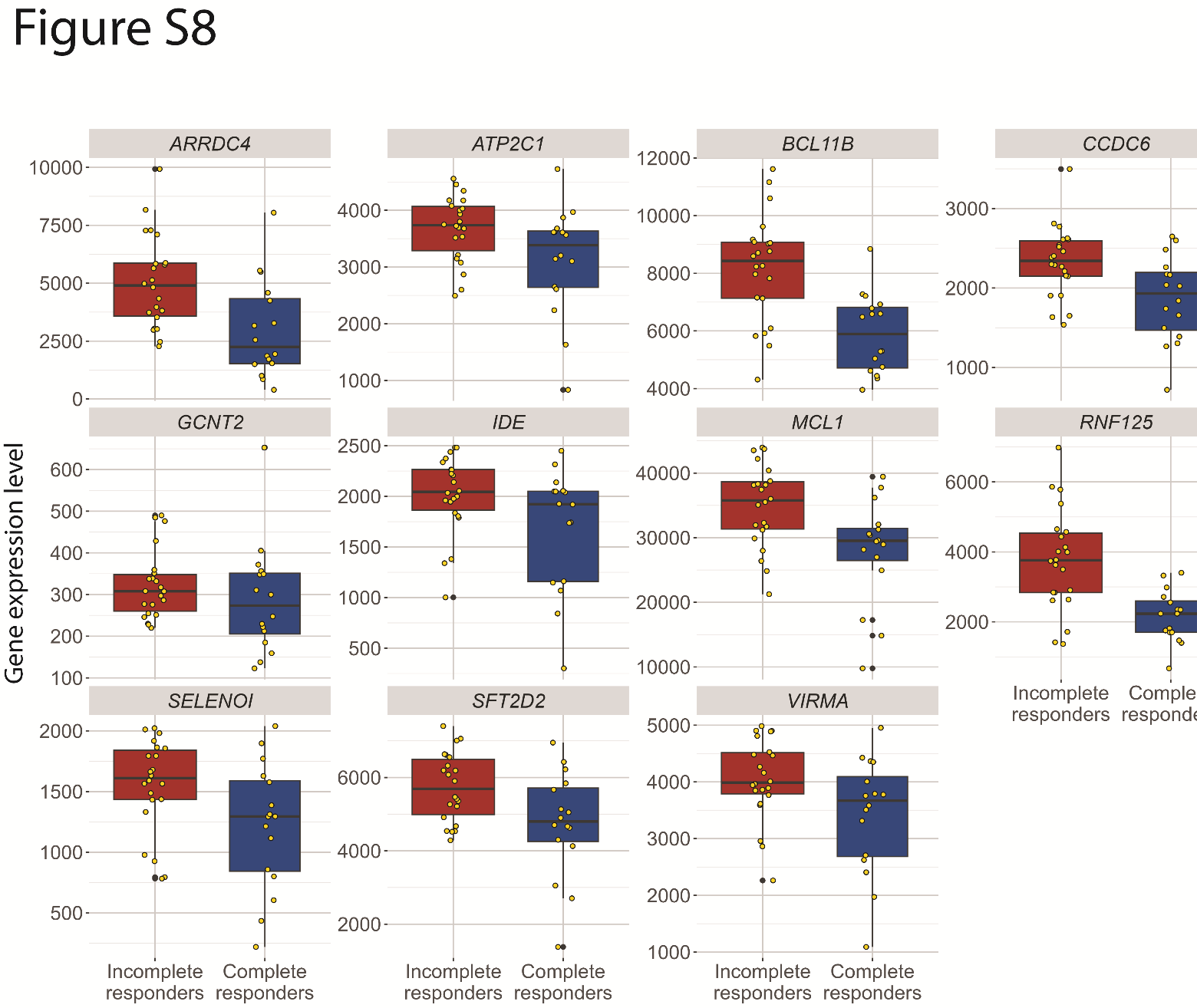


Figure S10. Expression levels of DEGs before OIT. Boxplots show raw gene expression levels of 11 significant DEGs in incomplete (red) and complete responders (dark blue), displaying the median and interquartile range. Individual values are shown as gold points. Abbreviations: DEGs, differentially expressed genes; *ARRDC4*, arrestin domain containing 4; *ATP2C1*, ATPase secretory pathway Ca2+ transporting 1; *BCL11B*, BCL11 transcription factor B; *CCDC6*, coiled-coil domain containing 6; DEGs, differentially expressed genes; *GCNT2*, glucosaminyl (N-acetyl) transferase 2; *IDE*, insulin degrading enzyme; *MCL1*, MCL1 apoptosis regulator; OIT, oral immunotherapy; *RNF125*, ring finger protein 125; *SELENOI*, selenoprotein I; *SFT2D2*, SFT2 Domain Containing 2; *VIRMA*, vir like m6A methyltransferase associated.


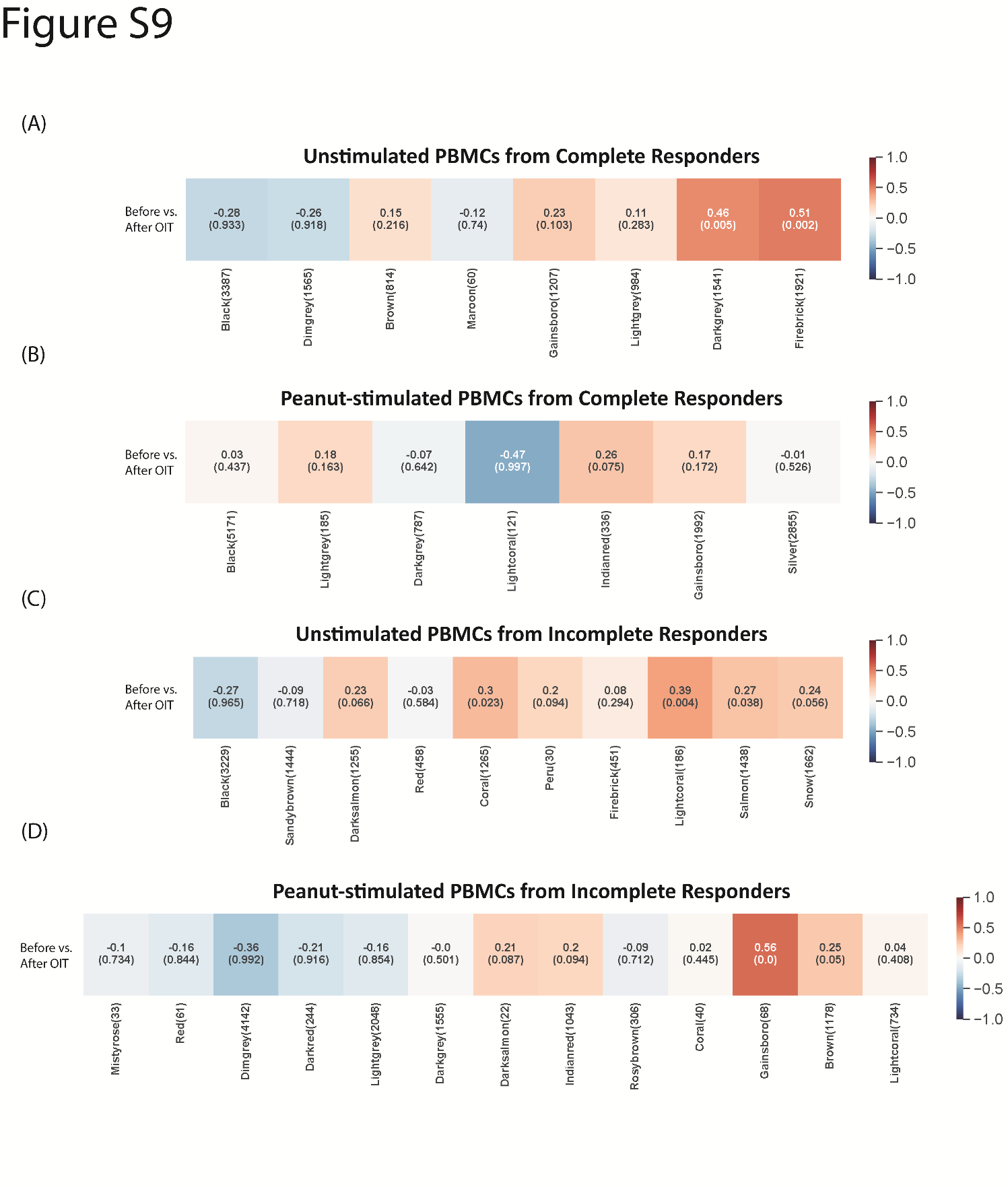


Figure S11. Module-trait relationship heatmaps illustrating correlations between WGCNA co-expression modules and sampling/OIT time points. The heatmaps depict Pearson correlations between co-expressed gene modules, identified by arbitrary color names (module sizes/number of genes indicated in parentheses), and sampling conditions comparing samples collected before and after OIT. Cell colors indicate the strength and direction of correlation (red, positive correlation, higher after OIT; blue, negative correlation, lower after OIT). Correlation coefficients are presented within each cell, with corresponding *p*-values in parentheses. Panels represent analyses stratified by stimulation condition and OIT outcome: (A) unstimulated PBMCs from complete responders; (B) peanut-stimulated PBMCs from complete responders; (C) unstimulated PBMCs from incomplete responders; and (D) peanut-stimulated PBMCs from incomplete responders. Abbreviations: WGCNA, weighted gene co-expression network analysis; OIT, oral immunotherapy; PBMCs, peripheral blood mononuclear cells.


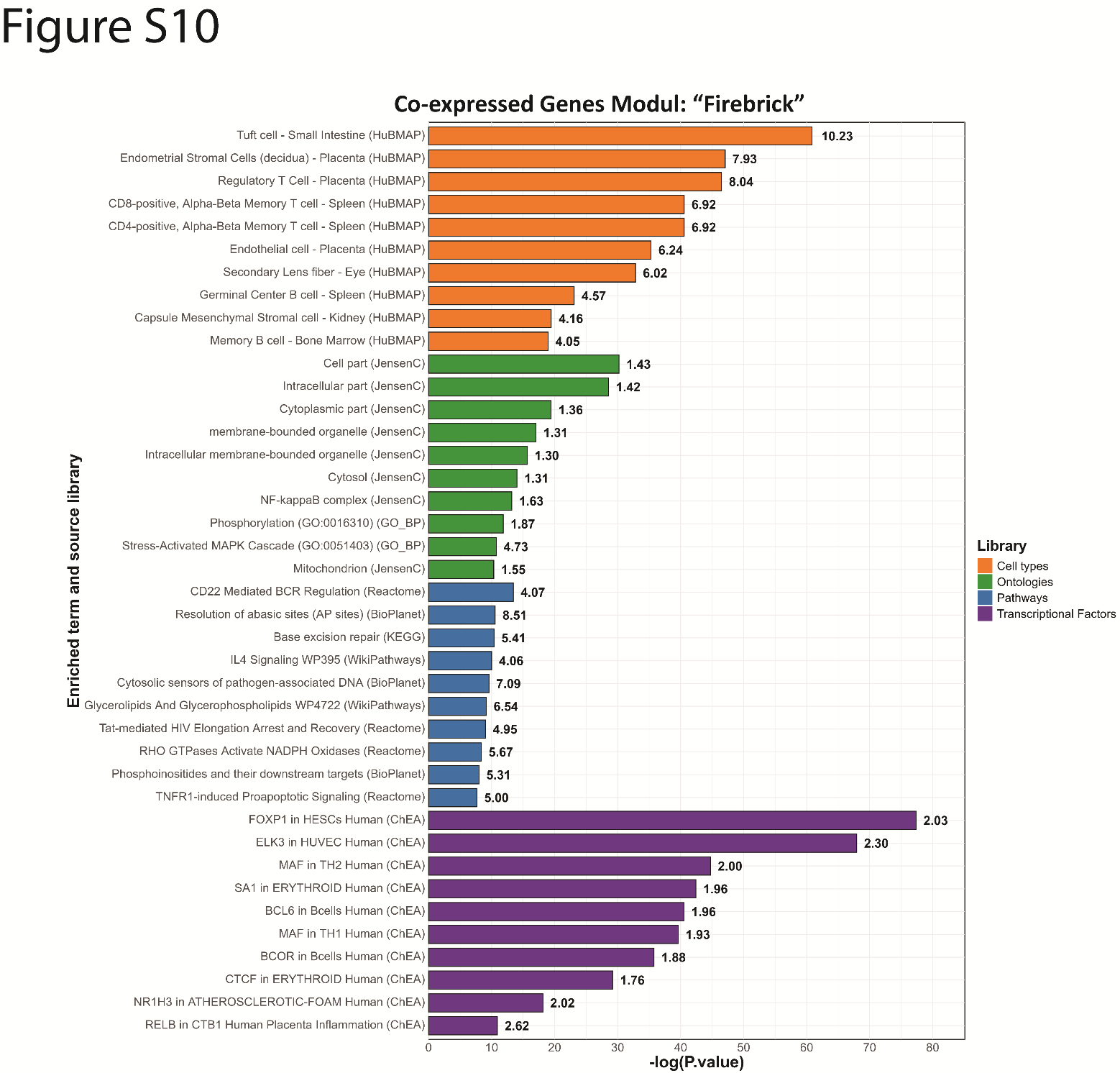


Figure S12. Functional enrichment analysis of the “Firebrick” gene module associated with changes before versus after OIT in unstimulated PBMCs from complete responders. The figure illustrates the top 10 enriched terms across four functional domains: Cell Types, Ontologies, Pathways, and Transcriptional Factors, identified through WGCNA of RNA-seq data from unstimulated PBMCs from complete responders. Terms are selected by odds ratio (top 10 highest values; shown in bold at the end of each bar) and subsequently sorted according to statistical significance (−log_10_ adjusted *p*-value). Extended results are provided in Supplementary Table S9. Abbreviations: OIT, oral immunotherapy; PBMCs, peripheral blood mononuclear cells; WGCNA, weighted gene co-expression network analysis; RNA-seq, RNA sequencing; HuBMAP, Human BioMolecular Atlas Program; JensenC, Jensen COMPARTMENTS; GO_BP, Gene Ontology Biological Process; KEGG, Kyoto Encyclopedia of Genes and Genomes; Reactome, Reactome Pathway Database; BioPlanet, NIH BioPlanet Pathway Database; WikiPathways, WikiPathways Pathway Database; ChEA, ChIP-X Enrichment Analysis.


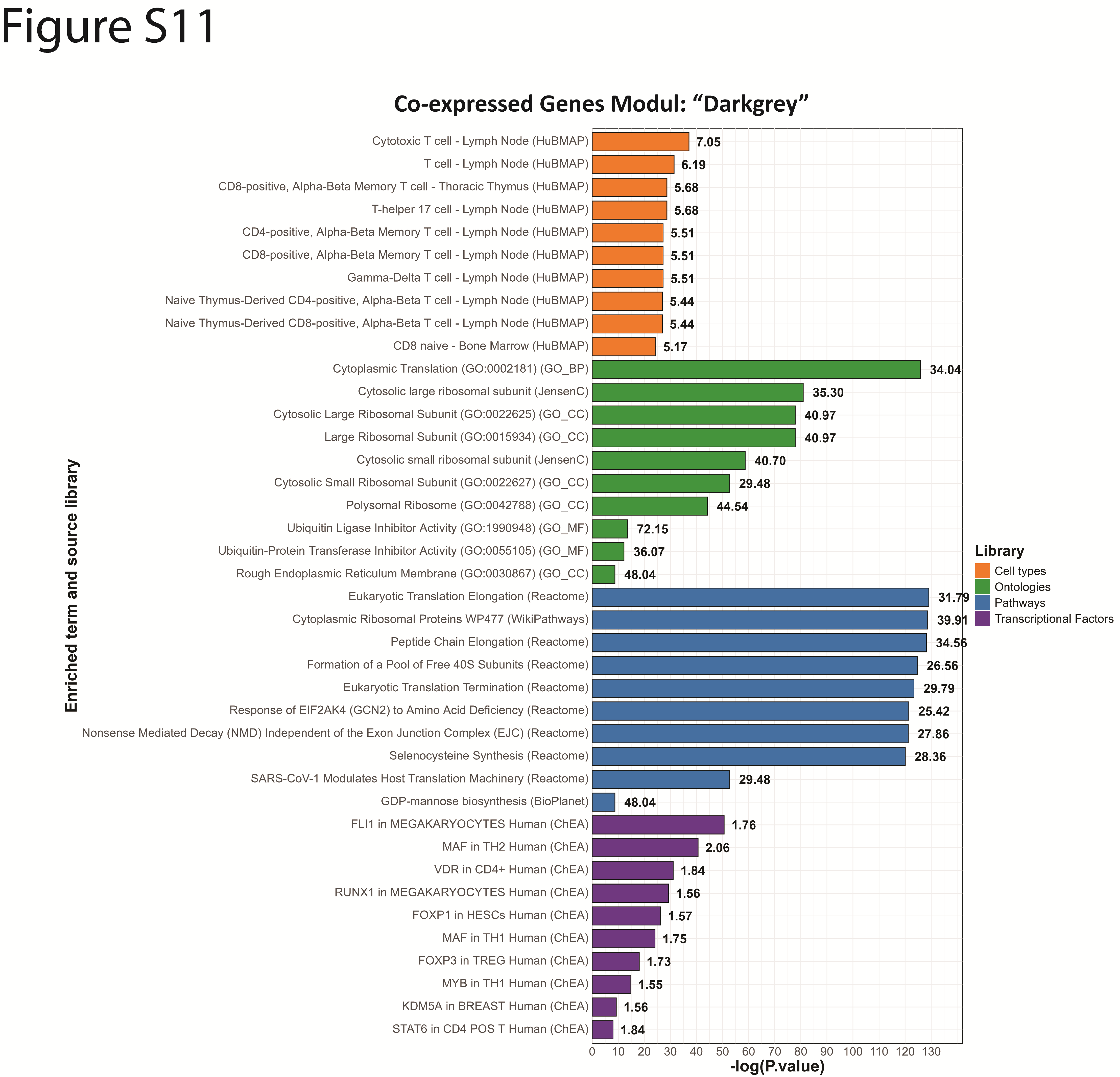


Figure S13. Functional enrichment analysis of the “Darkgrey” gene module associated with changes before versus after OIT in unstimulated PBMCs from complete responders. he figure illustrates the top 10 enriched terms across four functional domains: Cell Types, Ontologies, Pathways, and Transcriptional Factors, identified through WGCNA of RNA-seq data from unstimulated PBMCs from complete responders. Terms are selected by odds ratio (top 10 highest values; shown in bold at the end of each bar) and subsequently sorted according to statistical significance (−log_10_ adjusted *p*-value). Extended results are found in supplementary table S10. Abbreviations: OIT, oral immunotherapy; PBMCs, peripheral blood mononuclear cells; WGCNA, weighted gene co-expression network analysis; RNA-seq, RNA sequencing; HuBMAP, Human BioMolecular Atlas Program; JensenC, Jensen COMPARTMENTS; GO_BP, Gene Ontology Biological Process; KEGG, Kyoto Encyclopedia of Genes and Genomes; Reactome, Reactome Pathway Database; BioPlanet, NIH BioPlanet Pathway Database; WikiPathways, WikiPathways Pathway Database; ChEA, ChIP-X Enrichment Analysis.


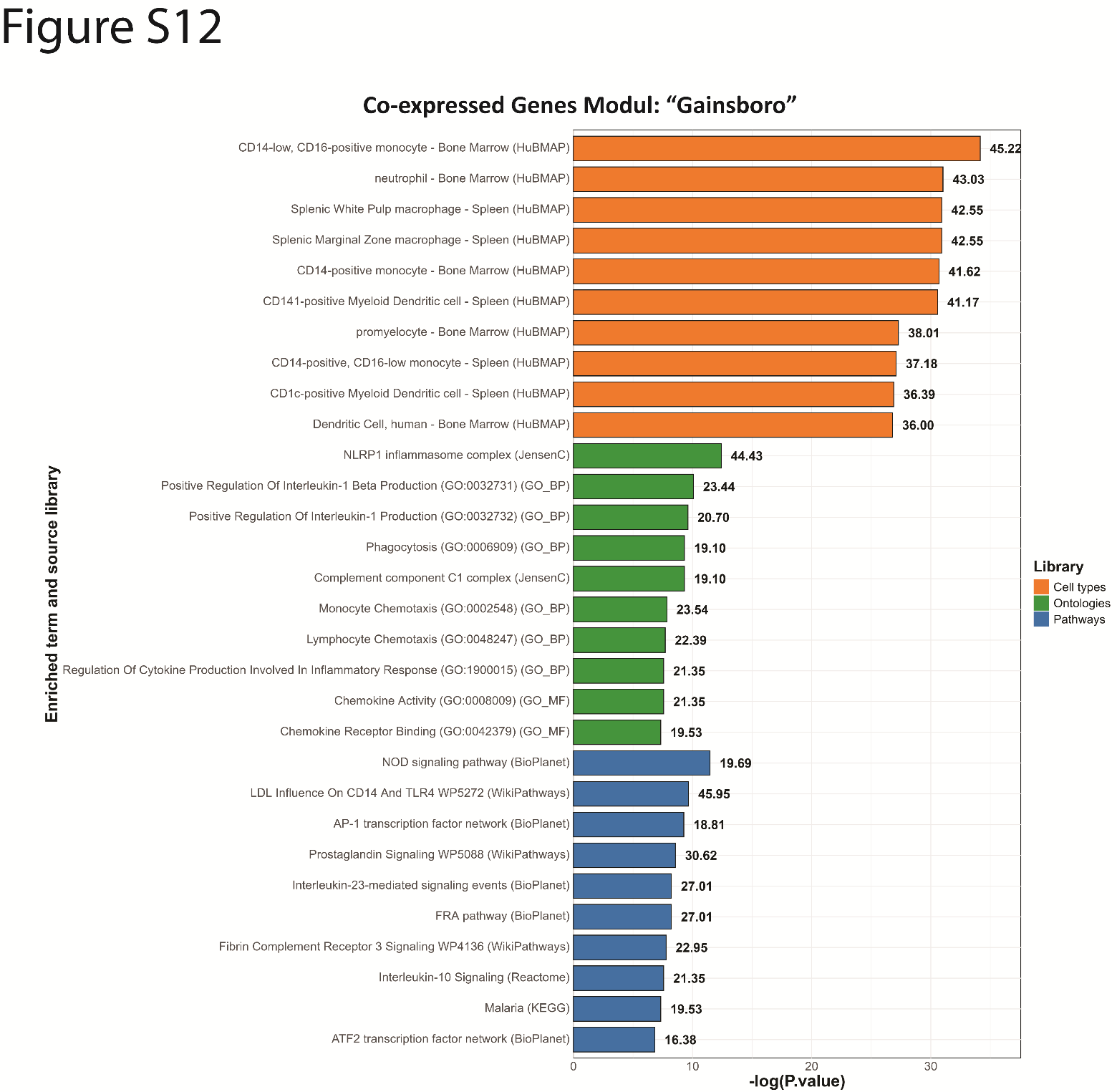
Figure S14. Functional enrichment analysis of the “Gainsboro” gene module associated with changes before versus after OIT in peanut-stimulated PBMCs from incomplete responders. The figure illustrates the top 10 enriched terms across three functional domains: Cell Types, Ontologies, and Pathways, identified through WGCNA of RNA-seq data from peanut-stimulated PBMCs from incomplete responders. Terms are selected by odds ratio (top 10 highest values; shown in bold at the end of each bar) and subsequently sorted according to statistical significance (−log_10_ adjusted *p*-value). Extended results are provided in Supplementary Table S11. **Abbreviations:** OIT, oral immunotherapy; PBMCs, peripheral blood mononuclear cells; WGCNA, weighted gene co-expression network analysis; RNA-seq, RNA sequencing; HuBMAP, Human BioMolecular Atlas Program; JensenC, Jensen COMPARTMENTS; GO_BP, Gene Ontology Biological Process; GO_MF, Gene Ontology Molecular Function; KEGG, Kyoto Encyclopedia of Genes and Genomes; Reactome, Reactome Pathway Database; BioPlanet, NIH BioPlanet Pathway Database; WikiPathways, WikiPathways Pathway Database.


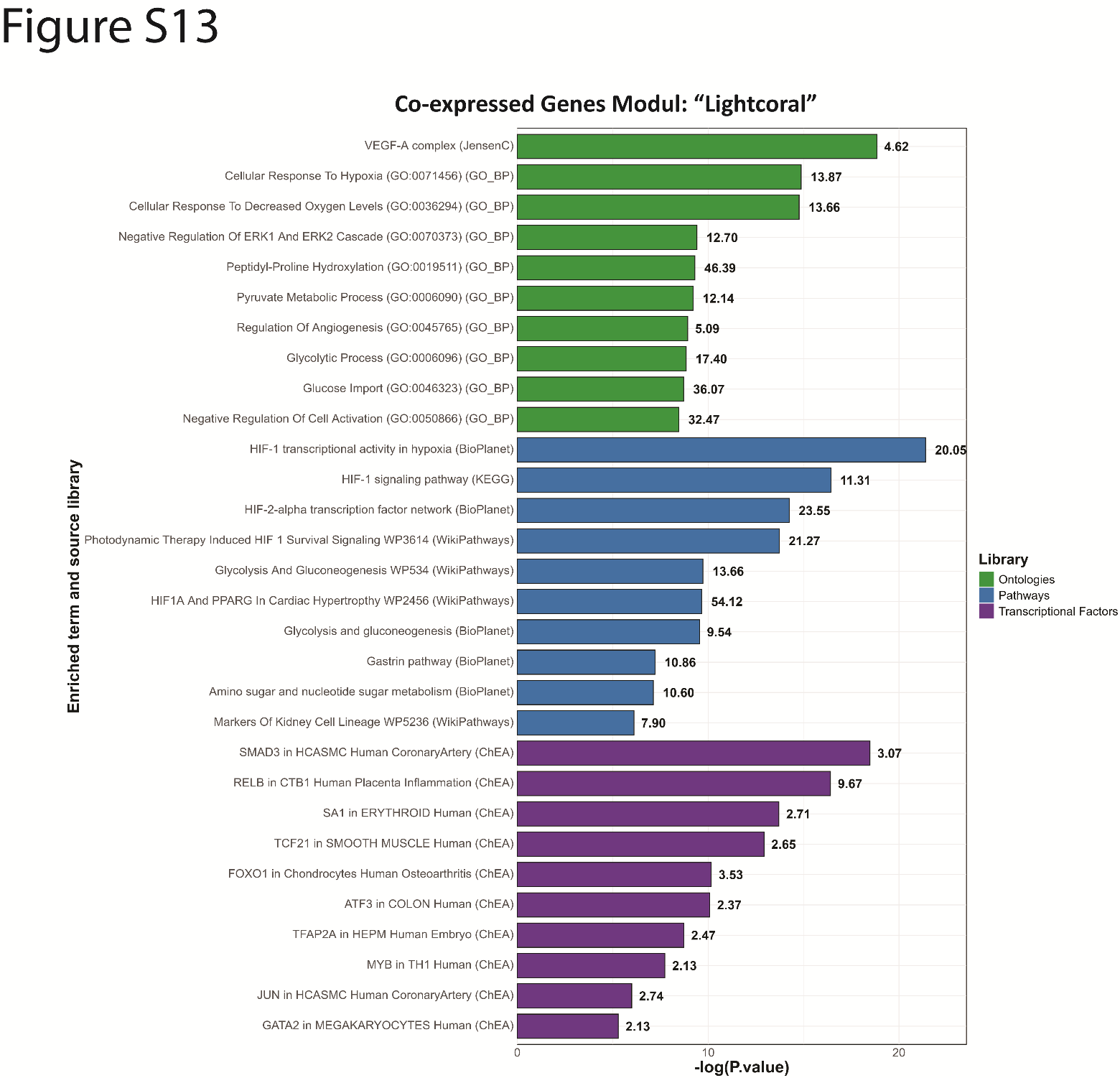


Figure S15. Functional enrichment analysis of the “Lightcoral” gene module associated with changes before versus after OIT in unstimulated PBMCs from incomplete responders. The figure illustrates the top 10 enriched terms across three functional domains: Ontologies, Pathways, and Transcriptional Factors, identified through WGCNA of RNA-seq data from unstimulated PBMCs from incomplete responders. Terms are selected by odds ratio (top 10 highest values; shown in bold at the end of each bar) and subsequently sorted according to statistical significance (−log_10_ adjusted *p*-value). Extended results are provided in Supplementary Table S12. **Abbreviations:** OIT, oral immunotherapy; PBMCs, peripheral blood mononuclear cells; WGCNA, weighted gene co-expression network analysis; RNA-seq, RNA sequencing; JensenC, Jensen COMPARTMENTS; GO_BP, Gene Ontology Biological Process; KEGG, Kyoto Encyclopedia of Genes and Genomes; BioPlanet, NIH BioPlanet Pathway Database; WikiPathways, WikiPathways Pathway Database; ChEA, ChIP-X Enrichment Analysis.
